# Mef2 Interacts with the Notch Pathway during Adult Muscle Development in *Drosophila melanogaster*

**DOI:** 10.1371/journal.pone.0108149

**Published:** 2014-09-23

**Authors:** Charlotte Caine, Petar Kasherov, Joël Silber, Alexis Lalouette

**Affiliations:** Institut Jacques Monod, CNRS, UMR 7592, Université Paris Diderot, Sorbonne Paris Cité, Paris, France; CNRS UMR7622 & University Paris 6 Pierre-et-Marie-Curie, France

## Abstract

Myogenesis of indirect flight muscles (IFMs) in *Drosophila melanogaster* follows a well-defined cellular developmental scheme. During embryogenesis, a set of cells, the Adult Muscle Precursors (AMPs), are specified. These cells will become proliferating myoblasts during the larval stages which will then give rise to the adult IFMs. Although the cellular aspect of this developmental process is well studied, the molecular biology behind the different stages is still under investigation. In particular, the interactions required during the transition from proliferating myoblasts to differentiated myoblasts ready to fuse to the muscle fiber. It has been previously shown that the Notch pathway is active in proliferating myoblasts, and that this pathway is inhibited in developing muscle fibers. Furthermore, the Myocyte Enhancing Factor 2 (Mef2), Vestigial (Vg) and Scalloped (Sd) transcription factors are necessary for IFM development and that Vg is required for Notch pathway repression in differentiating fibers. Here we examine the interactions between Notch and Mef2 and mechanisms by which the Notch pathway is inhibited during differentiation. We show that Mef2 is capable of inhibiting the Notch pathway in non myogenic cells. A previous screen for Mef2 potential targets identified *Delta* a component of the Notch pathway. *Dl* is expressed in Mef2 and Sd-positive developing fibers. Our results show that Mef2 and possibly Sd regulate a *Dl* enhancer specifically expressed in the developing IFMs and that Mef2 is required for Dl expression in developing IFMs.

## Introduction

Muscle development is a complex program that is evolutionarily well conserved. Muscle precursor cells are specified, then proliferate and fuse to form multinucleated myotubes which give rise to differentiated muscle fibers. The cellular changes that characterize this process have been well described whereas the molecular aspects have yet to be completely elucidated. *Drosophila melanogaster* has been shown to be a good model for understanding myogenesis, especially considering the conservation of the mechanism between *Drosophila* and mammals [1], [2].

Two waves of myogenesis have been described in *Drosophila*: the first occurs during embryogenesis to give rise to the larval muscles (for review, see [3], [4]), the second takes place during pupariation and leads to the adult muscles (for review, see [4]). A particular set of adult muscle structures, the Indirect Flight Muscles (IFMs) provide a valuable tissue context to study muscle development. IFMs are composed of 7 dorsal ventral muscles (DVMs) and 6 dorsal longitudinal muscles (DLMs) per hemithorax [5]. They develop between 8 and 36 hours after puparium formation (APF) from adult muscle precursor cells (AMPs) named myoblasts that have been specified during embryogenesis and have proliferated on the wing imaginal disc during larval stages [6], [7]. Interestingly, whereas DVMs are formed *de novo* during pupariation by myoblast fusion, DLMs are formed through fusion of myoblasts to larval scaffolds that escape histolysis [6].

The maintenance of the AMPs during the larval stages in a proliferative state requires the expression of the Twist bHLH transcription factor (Twi) and the activation of the Notch pathway. Twi has been primarily described for its role during mesoderm development in the embryo [8]. It is activated by the Notch pathway in AMPs and acts as an anti-differentiation signal (Figure S1, A) [9]. Interestingly, Twi activates the transcription of the *Myocyte Enhancer Factor 2* gene (*Mef2*) in AMPs [10]. Mef2 is a transcription factor essential for cardiac, visceral and somatic muscle development in the *Drosophila* embryo [11], [12]. Furthermore, in the adult fly, *Mef2* mutants show severe defects in IFM differentiation [13], [14] and *Mef2* overexpression in AMPs induces early differentiation, suggesting that Mef2 is the major differentiation factor in IFM development [15]. Consistent with this, Mef2 expression levels increase throughout IFM development, starting in the AMPs and reaching its maximal levels in the differentiating fibers [16]. Thus, the situation may appear paradoxical as Twi, the main anti-differentiation factor, activates the transcription of Mef2, the main pro-differentiation factor. In fact, while *Mef2* is expressed and Mef2 protein is present in AMPs, its transcriptional activity is repressed. Indeed, Twi and Notch together activate the *Holes in muscle* gene (*Him*) that encodes a repressor of Mef2 transcriptional activity [17]–[19]. Therefore, maintenance of AMPs in an undifferentiated state requires a subtle equilibrium directed by Notch between Mef2 quantity and its transcriptional activity. This predicts that differentiation could be triggered by a change in this equilibrium, either by over-activating Mef2 or by inhibiting the Notch pathway.

The Notch pathway is an evolutionarily conserved intercellular signaling pathway involved in numerous developmental processes such as cell-fate determination, neural development, and tissue homeostasis [20], [21]. In *Drosophila*, a cell that expresses one of the pathway transmembrane ligands, Delta (Dl) or Serrate (Ser), signals an adjacent cell expressing the transmembrane receptor Notch. Notch affinity for Dl and Ser is modulated by the glycosyltransferase Fringe (Fng): glycosylation of Notch increases its affinity for Dl and decreases its affinity for Ser [22]–[24]. The interaction of Notch with its ligands induces two proteolytic cleavages of Notch leading to the release of its intracellular domain (Nicd). The Nicd fragment translocates to the nucleus where it binds to its transcription cofactor Suppressor of Hairless (Su(H)) to regulate its target genes [20], [21], [25]–[30].

In differentiating IFMs, the Notch pathway is inhibited [9]. Twi and Him are not expressed and Mef2 is transcriptionally active [9], [19] (Figure S1, B). Moreover, ectopic activation of the Notch pathway in differentiating IFMs is sufficient to activate *twi* and *Him* and to induce muscle degeneration, showing that the pathway must be inactivated to allow differentiation [9], [19]. Thus, Notch inhibition is a key step of IFM differentiation. However, little is known about the mechanisms responsible for it. We know that Fng and the transcription factor Vestigial (Vg) are implicated [31]. *vg* was first described by its role during wing development [32]. *vg* product is devoid of a DNA binding domain and is capable of activating transcription of its targets when associated with its cofactor Scalloped (Sd). Sd can bind DNA but does not possess a transactivation domain [33]–[35]. Sd-Vg dimer plays an important role during wing, neural and muscle development [31], [36]–[38]. Indeed, *sd* is expressed in AMPs during larval stages and *vg* is expressed in a subset of AMPs during embryogenesis and larval stages; they are both involved in muscular identity [38]–[40]. Moreover, in a *vg^null^* context, IFM differentiation is severely impaired, Notch is ectopically activated in developing fibers and *fng* expression is lost in IFMs [31], [39]. Thus, in order for IFM differentiation to proceed, the anti-differentiation role of the Notch pathway must be repressed by Vg.

Altogether, data show that Sd, Vg and Mef2 are involved in IFM development. Moreover, Sd and Vg can both interact with Mef2 and Mef2 synergizes with Sd to activate a differentiation specific enhancer of *vg* [38], [41]. Interestingly, mammalian orthologs of Sd and Mef2 interact to activate muscle-specific enhancers, suggesting that the roles of Sd/Mef2 during myogenesis could be conserved between different species [42], [43]. In this study, we were interested in the mechanisms triggering Notch inhibition. If Vg is required, it is not sufficient and cannot be the triggering signal since it is expressed in AMPs where Notch is active [44]. We therefore focused on Mef2 for two reasons: i) Mef2 is capable of inducing differentiation [15], ii) Mef2 levels increase during development and its transcriptional targets depend on its activity level [16], [19], [45]. Here we show that Mef2 can repress the Notch pathway in various contexts. We propose that an aspect of this inhibition requires transcriptional regulation of *Delta*, a component of the Notch pathway. Indeed, we identified and characterized a 2.64 kb enhancer of *Delta* containing Mef2 and Sd binding sites and show that Mef2 is implicated in *Delta* regulation in the developing IFM muscle fibers. These results are consistent with the idea that Mef2 could repress the Notch pathway through *Dl* regulation.

## Materials and Methods

### Fly stocks

The following strains were used in this study: *UAS-Mef2* [11], *UAS-sd* [33], *1151-Gal4* [9], *UAS-H2B::YFP* [46], *UAS-Mef2[RNAi]* [47], *UAS-N^DN^* [48], *neur^p72^-Gal4* [46], *sd^11L^* [49], *sd^68L^* [49], *patched-Gal4* (Bloomington #2017). The *UAS-H2B::YFP*; *tub*-*Gal80^ts^, neuralized^P72^-Gal4*/SM5CyO, TM6Tb line was donated by Michel Gho.

### Over-expression in bristle cell lineage

*UAS-H2B::YFP*; *tub*-*Gal80^ts^, neuralized^P72^-Gal4*/SM5CyO, TM6Tb males were crossed to *UAS-Mef2*, *UAS-Mef2-UAS-sd*, or *UAS-N^DN^* females at 18°C. White pupae (0 hours after pupa formation, APF) were transferred to 29°C to allow GAL4 activity.

### Immunostaining and antibodies

Anti-Twi and anti-Mef2 were generously donated by S. Roth, and B. Paterson respectively, and used at 1∶200 and 1∶5000 dilutions respectively. Chicken anti-GFP (1∶1000 dilution) antibodies were purchased from AvesLab (Tigard, USA). Mouse anti-Cut, anti-Wingless, anti-Prospero and 22c10 were purchased from the Developmental Studies Hybridoma Bank, and were used at a 1∶200 (DSHB, University of Iowa, Department of Biology, Iowa City, IA 52242). Anti-Sens [50] was generously donated by H. Bellen and used at a 1∶3000 dilution. Anti-Vg (1∶200 dilution) was described in Goulev et al. [51]. Fluorescent-conjugated secondary antibodies were purchased either from Molecular Probes (Carlsbad, USA) or Jackson Immunoresearch (West Grove, USA) and used at a 1∶200 dilution. When needed, DAPI (Sigma-Aldrich, Saint-Louis, USA) was added with the secondary antibodies at 1 µg/ml concentration.

Pupae dissection was performed as previously described in Fernandes et al. [6]. Preparations were observed with a Zeiss 710 or Leica SP5 confocal microscope. Adult thoraxes were observed with a Keyence VHX-2000 microscope.

### Plasmid constructions and transgenesis

The *Dl2.6* sequence was amplified by PCR of genomic *Drosophila melanogaster* Canton S strain DNA (Oligonucleotides: D497_5_a2 5′CACTGGCGTATGCCACATCC3′ and D497_6_a2 5′-ACAAGGGCTTCACGAATCCC) and cloned into the pGEM-T *easy* vector (Promega, Madison, USA). *Dl2.6* was subcloned i) into the pGL3 plasmid with *Eco*RI and *Xba*I restriction enzymes (*Dl2.6-luc*) and ii) into the transgenesis pGreen-H-Pelican vector [52] with *Kpn*I-*Bgl*II and *Kpn*I-*Xho*I restriction enzymes respectively (*pGreen-H-Pelican*-*Dl2.6*). *pCasPeR-hsp70-Mef2* and *pCasPer-hsp70*-*sd* plasmids are described in Bernard et al. [41]. Transgenesis was performed by BestGene (Chino Hills, USA).

### Cell culture experiments and transfection assays

*Drosophila* S2 cells were maintained at 22°C in standard Schneider medium with fetal bovine serum (FBS 10% v/v) and antibiotics (streptomycin 100 µg/ml and penicillin 100 U/ml). *Drosophila* Dmd8 cells were also maintained at 22°C in standard Schneider medium with fetal bovine serum (FBS 5% v/v), antibiotics (streptomycin 100 µg/ml and penicillin 100 U/ml) and insulin (1%). Transfection assays were performed with Effectene reagent (QIAGEN, Valencia, USA) according to the manufacturer's instructions. The Effectene: DNA ratio was 10∶1 (µl∶µg). For normalization, the *pCasper-hsp*-*Actin-LacZ* plasmid (a gift from M. Sanial) was co-transfected. Cells were transfected with 1 µg of total plasmid DNA (100 ng of *pGL3-Dl2.6*, 100 ng of *pCasper-hsp-Actin-LacZ* vector, 100 to 300 ng of each expression vector (*pCasPer-hsp70-Mef2* or *pCasPer-hsp70*-*sd*) adjusted to 1 µg with empty *pCasPer-hsp70* vector).

Following incubation for 24 h at 22°C, cells were heat-shocked at 37°C for 1 h and incubated for an additional 24 h at 22°C. Firefly luciferase activities were assayed with the Luciferase Assay System kit (Promega Madison, USA). β-Galactosidase activity was measured with ONPG assays as previously described [53]. In each experiment, the mean and standard deviation were calculated on six independent transfection assays. Means were compared using the Student's *t*-test.

### Q-PCR experiments

Transfection assays were carried out in Dmd8 cells using 500 ng of each expression vector (*pCasPer-hsp70-Mef2* or *sd* adjusted to 1 µg with empty *pCasPer-hsp70* vector). RNA extractions were done using the RNAeasy kit (QIAGEN, Valencia, USA). 100 ng of RNA from each extraction were retrotranscribed using the Superscript III VILO (Life Technologies, Paisley, UK) according to the manufacturer's protocol. Quantitative RT-PCR amplification mixture (10 µl) contained 1/20 of cDNA product, 10X SYBR Green I Master Mix buffer and 100 ng forward and reverse primers. The expression of *Dl* was quantified relative to *rp49* housekeeping gene [54]. Each point was repeated three times. Primers for *rp49* amplification were: forward primer 5'-CCGCTTCAAGGGACAGTATCTG, reverse primer: 5'-CACGTTGTGCACCAGGAACTT.

*Dl* amplification was carried out with: forward primer 5′ -CCAGCGACTCTTGGTGCAGC, reverse primer 5′ -GTGGCCTGGTAGTGCTTTAGG. Reactions were run on a Light Cycler PCR machine (Roche). Cycle condition were 10 min at 95°C and 45 cycles at 95°C for 10 sec, 60°C (*RP49, Dl*) for 10 sec and 72°C for 10 sec. Quantification was done using the mathematical model described in Pfaffl et al. [55].

## Results

The published data have demonstrated that *vg and sd* are expressed throughout IFM development, yet Vg-Sd is required to inhibit the Notch pathway only late in the muscle differentiation program, in the developing fibers [39], [40]. This is not due to differential *vg* expression levels considering that neither overexpression nor hypomorphic mutants of *vg* display any muscle phenotypes (data not shown). This led us to hypothesize that another factor triggers IFM differentiation. Recent experiments have shown that Mef2 can interact physically with Vg and Sd [38], [41] and that Sd and Mef2 synergize to activate a differentiation-specific enhancer of *vg* (*vg^AME^*) in the differentiating IFM [41], indicating that Mef2 could be a good candidate for Notch pathway inhibition [14], [15], [56]–[60].

### Mef2 can repress the Notch pathway at the DV boundary of the wing disc

**Figure 1:**
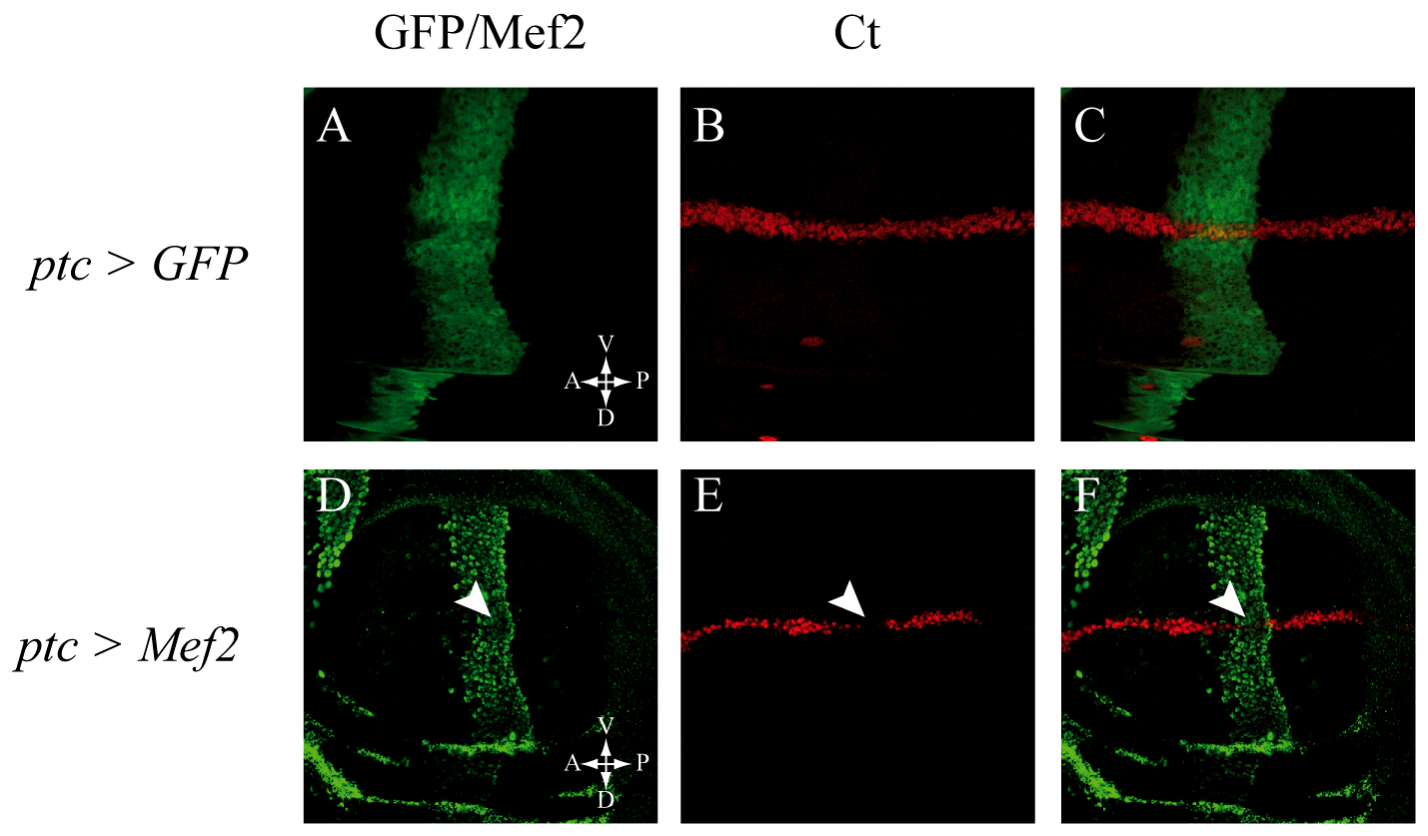
Repression of Notch signaling by Mef2 at the DV boundary of the wing disc. When GFP is expressed along the AP boundary of the third instar larva wing disc using the *ptc-Gal4* driver (A), Ct, detected by a specific anti-Ct antibody, is normally expressed at the DV boundary (B; overlay in C). In contrast, mis-expression of Mef2 along the AP boundary using the same driver (D; detected using an anti-Mef2 antibody) severely reduces Ct expression at the AP boundary (E, arrowhead; overlay in F).

Our first goal was to test whether Mef2 by itself can inhibit the Notch pathway. However, it has been shown that Mef2 overexpression in myogenic cells induces premature muscle differentiation [19], hence making it difficult to see whether Notch pathway repression could be the consequence of Mef2 expression or the consequence of the myogenic differentiation program. To circumvent this problem, we ectopically misexpressed Mef2 in non-myogenic cells in which the Notch pathway is activated. The Notch pathway is active in a number of developmental processes and has different transcriptional outputs depending on the cellular context. These outputs can be used to determine the activity of the pathway [25], [26], [29], [30], [61]. At the dorsal-ventral (DV) boundary of the wing imaginal disc, the Notch signaling induces *cut* (*ct*) expression [62]. We asked if Mef2 can repress the Notch pathway in the wing disc, by testing if its mis-expression can prevent expression of *ct*. Normally (in *ptc-gal4; UAS-GFP* flies), Ct is expressed along the DV boundary (Fig. 1A–C). Mis-expression of Mef2 along the AP boundary using the *ptc-gal4* driver (Fig. 1E), results in loss of Ct expression in the cells that ectopically express Mef2 (Fig. 1D, overlay in 1F). Two other Notch targets, Vg and Wingless (Wg) were tested and again a clear decrease in their expression was observed in Mef2 misexpressing cells (Figure S2). Thus, Mef2 represses Notch pathway activity in non-myogenic cells at the DV boundary of the wing disc. However, data showed that Mef2 can interact with Sd during myogenesis to activate muscle genes or enhancers [38], [41]. As Sd is expressed in the wing pouch [63], we cannot exclude that Notch repression at the DV boundary is due to interactions between Mef2 and endogenous Sd.

### Mef2 can inhibit the Notch pathway in the sensory organ lineage

The repression of Notch targets by Mef2 at the DV boundary of the wing disc could be due either to an interaction of Mef2 with the Notch pathway or to a change in cell identity, making Mef2 overexpressing cells non responsive to the Notch signal. In order to discriminate between these two possibilities, we examined another tissue context in which the Notch pathway is active and in which we could identify cell identity. Therefore, we decided to test Mef2 ability to repress the Notch pathway in the sensory organ lineage. As Mef2 could interact with Sd to repress the Notch pathway, we decided to perform Sd, Mef2 and Sd/Mef2 overexpressions.

The sensory organs of *Drosophila* are chaetes or bristles that are located on various parts of the body: the head, thorax and legs. Each organ is composed of a group of four cells: the neuron, the sheath cell, the shaft cell and the socket cell (for review, see [64]). The Notch pathway is necessary for lateral inhibition that takes place during the larval stages to determine the pI cell (for review, see [21]). This cell undergoes a first asymmetric division to give rise to the pIIa cell in which the Notch pathway is active (N^on^) and the pIIb cell where it is not (N^off^; Figure S3). The pIIb cell expresses Prospero (Pros) whereas the pIIa does not [65], [66]. Asymmetric division of the pIIa cell then gives rise to the shaft and socket cells where the Notch pathway is only active in the socket cell. The pIIb cell undergoes an asymmetric division that gives a glial cell and the pIIIb cell with an active Notch pathway. Finally, the pIIIb cell divides asymmetrically to give rise to the sheath cell in which the Notch pathway is active and the neuron.

**Figure 2:**
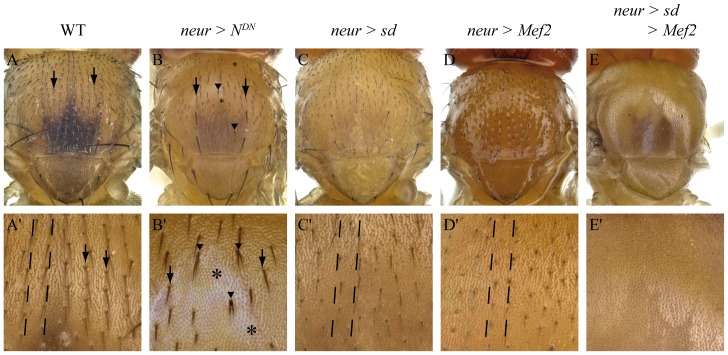
Notch repression by Sd and Mef2 in SOPs. A'–E' are magnifications of A–E. In wild type thoraxes, bristles (A, A' arrows) are aligned along the antero-posterior axis (A', dashed lines). When a dominant negative allele of *Notch* is expressed using the *neur-Gal4* driver, different Notch phenotypes are observed on thoraxes, spanning from wild-type bristles (B, B', arrows), to complete absence of external cells, shaft and socket (B, B', asterisks). Duplicated bristles resulting from Notch inhibition during the pI/pII or pII/pIII asymetric divisions are also present (B', arrowheads). When *sd* (C, C') or *Mef2* (D, D') are expressed alone, no *Notch* phenotypes are observed: bristles are present and align along the antero-posterior axis (C', D', dotted line). When *sd* and *Mef2* are expressed together, no external cells are observed, corresponding to a strong *Notch* phenotype (E, E').

**Figure 3:**
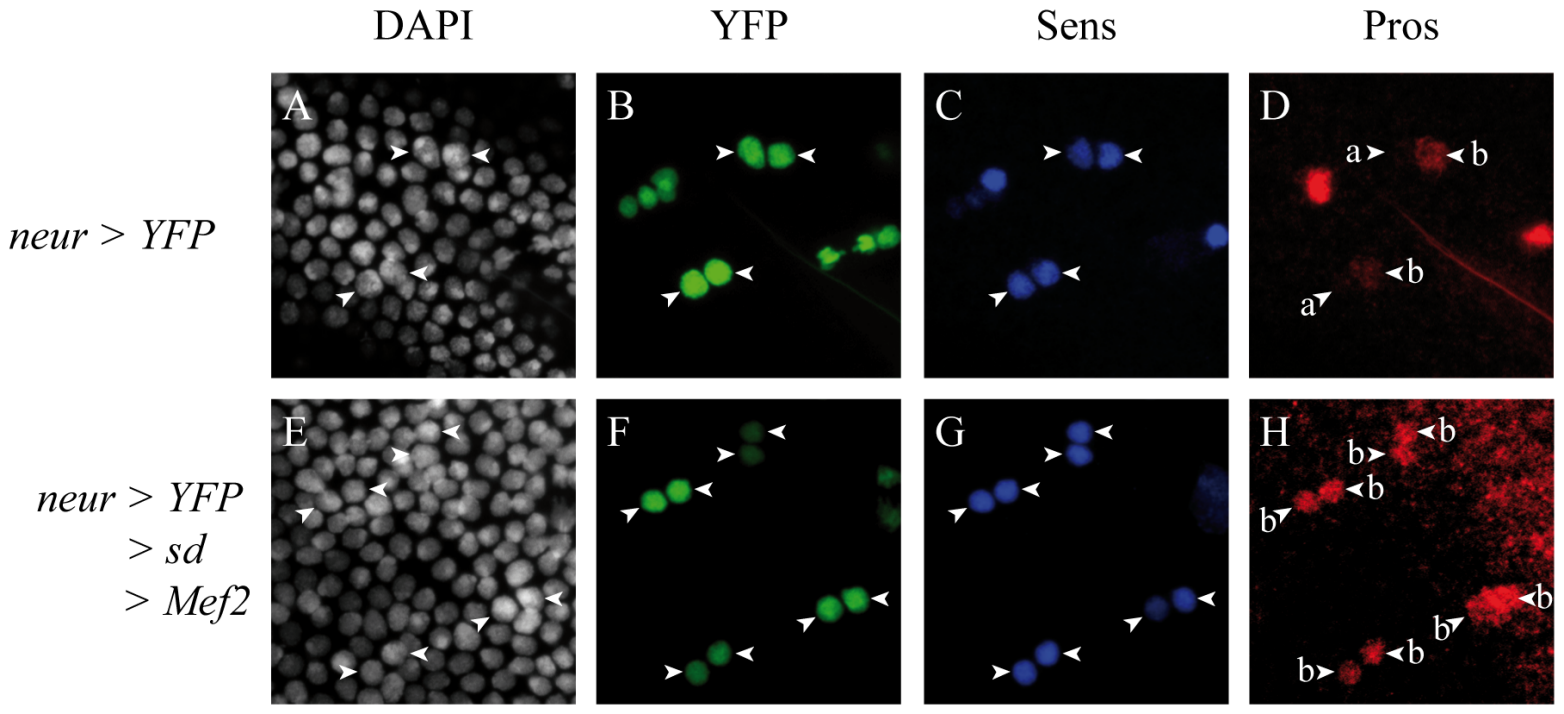
The Notch pathway is repressed during the first asymmetric division of SOPs upon Sd/Mef2 ectopic overexpression. YFP (A–D) or YFP, *sd*, and *Mef2* (E–H) were overexpressed using the *neur-Gal4* driver. In wild type genetic context, cells expressing the YFP (B) express Sens, a marker of the SOP lineage, detected with a specific antibody (C). In two cell clusters (A–D, arrowheads), only one cell corresponding to the pIIb cell expresses Pros (D, arrowhead b). The second cell corresponding to the pIIa cell does not express Pros (D, arrowhead a). When Sd, Mef2 and YFP are co-overexpressed, cells expressing YFP (F), as in wild type, express Sens (G) showing that these cells maintain their SOP identity. The cells of the two cell clusters (E–H, arrowheads) both express Pros (H, arrowhead b), signifying that they correspond to two pIIb cells. A and E correspond to DAPI staining.

In a wild-type adult thorax, small bristles (microchaetes) are present (Fig. 2A, A′, arrows), showing a specific distribution pattern, aligned along the antero-posterior axis (Fig. 2A′, dotted lines). Misexpression of a dominant-negative allele of Notch (*N^DN^*) between 0h and 30h APF in sensory organ precursors (SOPs) lineage using the *neur-Gal4* driver, leads to a strongly disorganized microcheate arrangement (Fig. 2B, B′). Patches with no external cells (shaft or socket) are observed (Fig. 2B, B′, asterisks). In some cases, duplications of the shaft are seen (Fig. 2B, B′ arrowheads). However, some microchaetes look normal, showing that the *N^DN^* allele is not fully penetrant (Fig. 2B, arrows). When *sd* or *Mef2* were misexpressed alone using the same driver, no Notch phenotype was observed even if some bristles seemed shorter: external cells are present, bristles are still organized along the antero-posterior axis, and no shaft duplications were observed (Fig. 2C, C′, D, D′, dotted lines). In contrast, when *sd* and *Mef2* were simultaneously misexpressed, no external cells were observed (Fig. 2E, E′), consistent with the idea that either that the pI cell is not specified or that the Notch pathway is inhibited after the pI cell specification, giving rise to two pIIb cells after cell division (see Figure S3). To test these two hypotheses, we looked at the developing nota at 21h APF. At this time point, in wild type flies (*neur-Gal4*; *UAS-H2B::YFP* genetic context) SOPs are already specified (Fig. 3B) and express the transcription factor Senseless, a marker of the SOP lineage (Sens, Fig. 3C, [50]). Moreover, as expected, in two cell clusters (Fig. 3A–D, arrowheads), one cell corresponding to the pIIb expresses Pros (Fig. 3D, arrowhead b) and the other cell corresponding to pIIa does not (3D, arrowhead a). When Sd and Mef2 are misexpressed (Fig. 3E–H) SOPs are specified and express Sens (Fig. 3F–G). In two cell clusters, the two cells express Pros, signifying that they all adopt a pIIb fate (Fig. 3H, arrowhead b). This phenotype is observed in Notch loss of function genetic background [66]. Therefore, when Sd and Mef2 are together misexpressed in SOPs, the Notch pathway is inhibited, at least during the first asymmetric division, and this repression is not due to a change in cell fate. This inhibition during first asymmetric division is consistent with phenotypes observed in adults. However, Mef2 or Sd alone overexpression do not show Notch-like adult phenotypes, suggesting that Sd and Mef2 are required to inhibit the Notch pathway.

### Identification of a putative Sd/Mef2 response element in the *Delta*

Our data show that Mef2 and probably Sd interact with the Notch pathway. As Sd/Mef2 acts as a transcription factor, we wondered whether Sd/Mef2 could directly activate genes involved in the Notch pathway. A previous study identified 670 genomic regions bound by Mef2 [67]. We decided to rescreen these 670 regions for clusters of Sd and Mef2 binding sites using the Cluster-Buster software (Cluster Buster, http://zlab.bu.edu/cluster-buster/). This led us to identify two overlapping genomic regions associated to the Notch pathway ligand *Dl* gene (clones D497_5_a2 and D497_6_a2; *mef2* ChIP data http://furlonglab.embl.de/). As *Dl* is expressed during IFM development [31] in the differentiating fibers, this makes it a potential Sd/Mef2 target.

### *Dl2.6* activation profile *in vivo*

In order to ask whether *Dl* could be regulated by Sd and/or Mef2, we generated transgenic fly lines in which 2640 bp *Dl* genomic fragment (*Dl2.6*) was used to drive GFP expression. *Dl2.6* sequence spans the two clones identified to be bound by Mef2 (see above) and containing 2 Sd and 9 Mef2 predicted binding sites (Figure S4).

**Figure 4:**
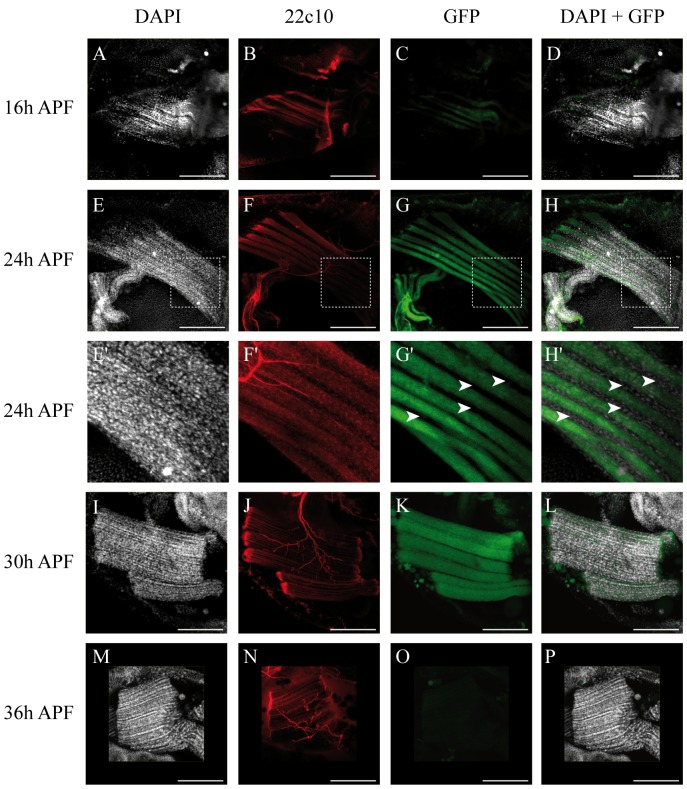
Activation of the *Dl2.6* enhancer *in vivo* monitored by the expression of the *Dl2.6-GFP* transgene. E′–H′ are magnifications of the dotted squares in E–H. In 16 h, 24 h, 30 h and 36 h APF pupae, the 22c10 antibody labels muscles and nerves (B, F, F′, J and N respectively), *Dl2.6* enhancer is activated in developing fibers of 16 h, 24 h and 30 h APF pupae (C, G, G′, K respectively) but not in myoblasts at 16 h APF (A–D) and 24 h APF (E–H, magnification in E′–H′, arrowheads). *Dl2.6* enhancer is not activated in developing fibers of 36 h APF pupa (M-P). DAPI-GFP overlay is shown in D, H, and H′, L and P. Scale bar: 100 µm.

**Figure 5:**
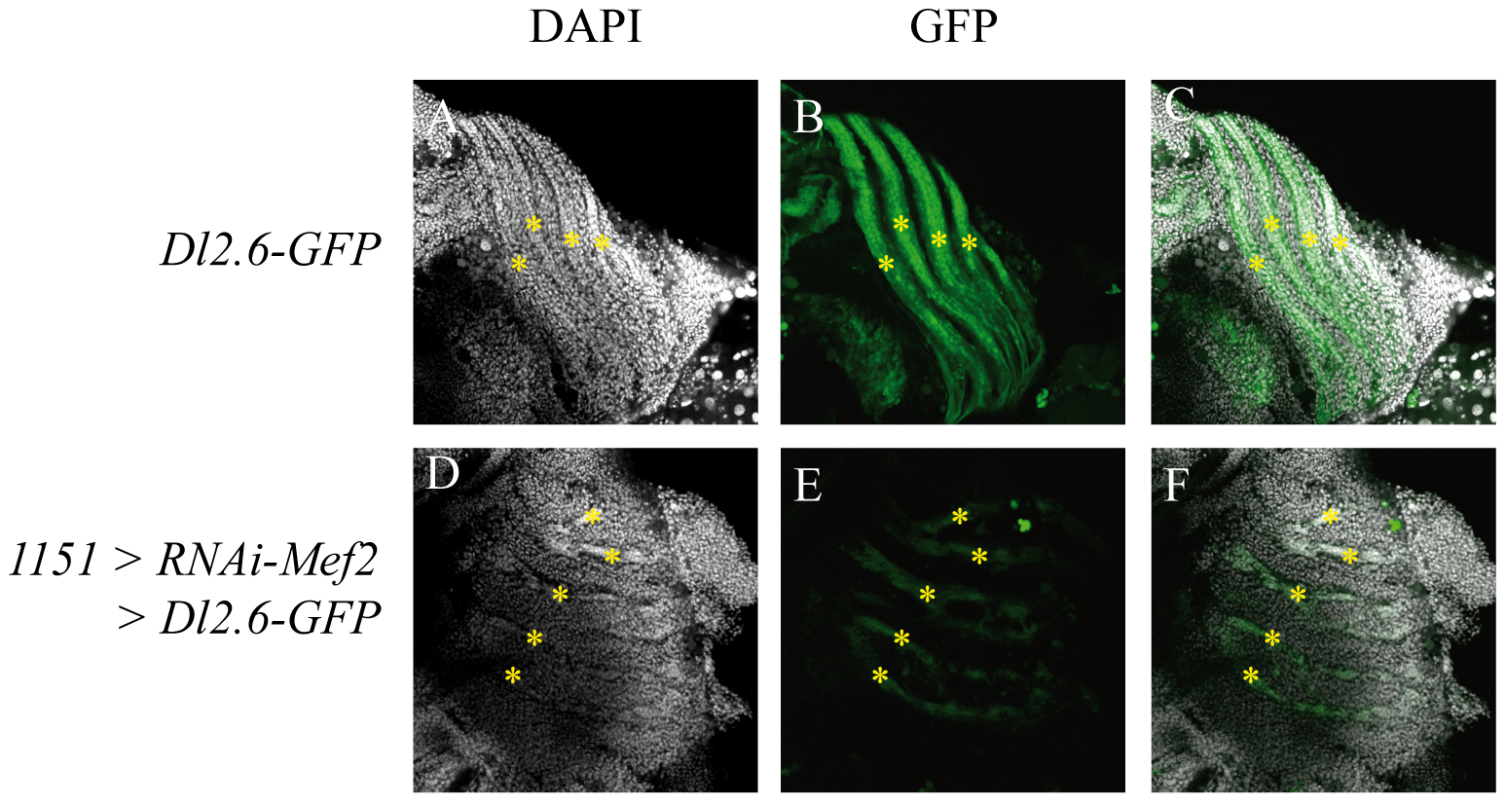
Reduced activation of the *Dl2.6* enhancer *in vivo* monitored by the expression of the *Dl2.6-GFP* transgene. In 21 h APF pupae, muscle fibers are visualized with DAPI staining by the specific arrangement of their nuclei (A, D, asterisks). In a wild-type pupae, *Dl2.6* enhancer is activated in developing fibers (B, overlay in C). In pupae overexpressing an *RNAi-Mef2* transgene, *Dl2.6* enhancer activation is significantly lower than in wild-type (E, overlay in F).

*Dl2.6-GFP* expression starts in developing fibers at 16 h APF (Fig. 4A–D). Activation persists in fibers at 24 h APF (Fig. 4E–H) and 30 h APF (Fig. 4I–L). No activation is detected in the swarming myoblasts around the fibers at 24 h APF (Fig. 4E′–H′, arrowheads) or during late differentiation stages from 36 h APF (Fig. 4M–P). In conclusion, the *Dl2.6* enhancer drives the GFP expression in developing DLMs during the early stages of their development (16 h–30 h APF). We next decided to test whether the *Dl2.6* enhancer is activated by Mef2 and Sd. When *Mef2* levels are decreased by overexpressing an *RNAi-Mef2* transgene in 21 h APF pupae (*1151-Gal4, UAS-RNAi-Mef2*), *Dl2.6* activation is lower than in wild-type genetic context (Fig. 5). This result shows that Mef2 is required *in vivo* for *Dl2.6* activation. As null *sd* mutants are early lethal, we decided to test *Dl2.6* activation in *sd^11L^* and *sd^68L^* pupal lethal alleles [49]. We observed a significant activation of *Dl2.6* in these genetic contexts (Figure S5) implying that either that Sd is not required or that these mutant alleles are not strong enough to interfere with *Dl2.6* activation.

### Regulation of *Dl2.6 in vitro*

**Figure 6:**
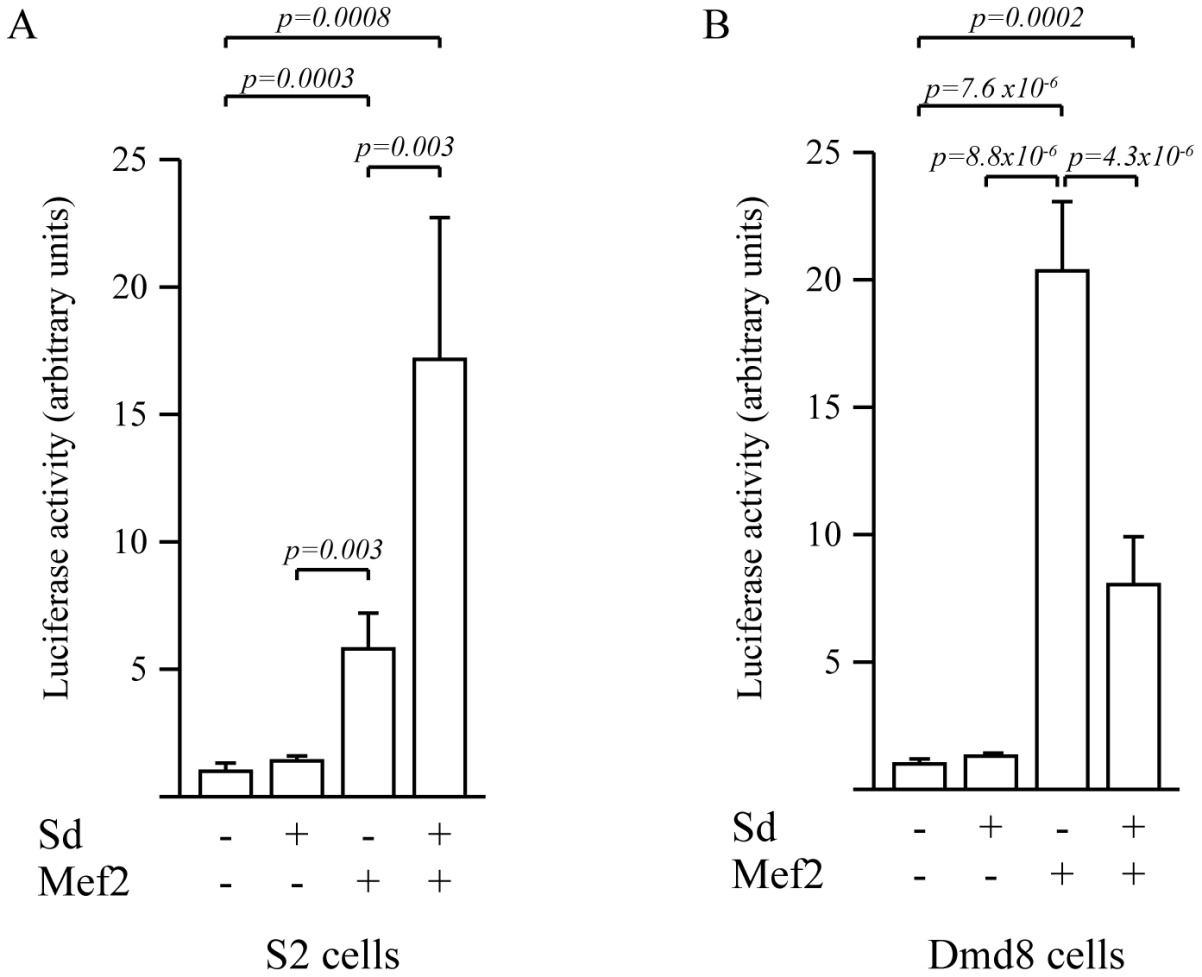
Activation of the *Dl2.6* enhancer in S2 and Dmd8 cells by Sd and Mef2. A: In S2 cells, the *Dl2.6* enhancer was not activated by Sd alone. In contrast, it was significantly activated by Mef2 (*p* = 0.0003) and Sd/Mef2 (*p* = 0.0008). Activation with Sd/Mef2 was significantly higher than activation by Mef2 alone (*p* = 0.003). B: In Dmd8 cells, as in S2 cells, *Dl2.6* is not activated by Sd alone. It is significantly activated by Mef2 (*p* = 7.6×10^−6^) and Sd/Mef2 (*p* = 2×10^−4^). However, activation by Sd/Mef2 was lower than activation by Mef2 alone (*p* = 4.3×10^−6^).

In order to examine its regulation, we cloned *Dl2*.6 in a luciferase reporter vector to measure its activity in cell culture experiments. We used two *Drosophila* cell lines to analyze enhancer activation by Sd and or Mef2, as both have putative binding sites located in the enhancer. The first is the classical Schneider 2 (S2) cell line, derived from a primary culture of embryonic *Drosophila* cells [68]. The second is the DmD8 cell line, derived from myoblasts located on the wing disc in the third instar larval stage of *Drosophila* development [17], [69], [70]. We co-transfected the *Dl2.6-luc* reporter plasmid with various combinations of Mef2 and Sd expression vectors (see Material and Methods) and quantified the luciferase activity. In S2 cells, the *Dl2.6* enhancer is not activated when Sd is transfected alone (Fig. 6A; Table S1). When Mef2 is overexpressed, we observe a significant activation (Fig. 6A, *p = 0.*003; Table S1). This activation is further substantially increased when Sd and Mef2 are overexpressed together (Fig. 6A, *p = 8×10^−4^* compared to control, *p = 3×10^−3^* compared to Mef2 overexpression; Table S1). In the Dmd8 cell line, the *Dl2.6* enhancer is also not activated by Sd alone. In contrast, it is significantly activated by Mef2 alone (Fig. 6B, *p = 7.6×10^−6^*; Table S2). When both Sd and Mef2 are present, this activation is significantly decreased relative to Mef2 alone (Fig. 6B, *p = 4.3×10^−6^*; Table S2), but still significantly higher than the baseline expression (Fig. 6B; compared to empty plasmids *p* = *2.3×10^−4^*; Table S2).

Our data show that Mef2 activates the *Dl2.*6 enhancer and that, in S2 cells, Mef2 synergizes with Sd for this activation. However, the level of activation by Sd/Mef2 in S2 cells is close to the level of activation by Mef2 alone in Dmd8 cells. We know that Dmd8 cells derive from 3^rd^ instar wing disc AMPs and are therefore likely to express *sd* [69]. Thus, this high activation is possibly due to interactions between endogenous Sd and transfected Mef2. A possible explanation for the reduced activation when Sd and Mef2 are together transfected in Dmd8 cells could be a “squelching” phenomenon [71]. Accordingly, in S2 cells where there is no Sd or Mef2, co-transfection of Mef2 and Sd shows a significantly increased activation compared to Mef2 alone.

We next determined whether this activation of the *Dl2.6* enhancer by Sd and Mef2 is associated with an increase of endogenous *Dl* mRNA levels. We quantified *Dl* mRNAs by Q-RT-PCR in Dmd8 cell culture experiments. When Dmd8 cells are transfected by *Mef2* and *sd* expressing plasmids, we observed a significant increase in *Dl* mRNA levels (2.32 fold, 5% confidence interval: [1.66–3.23]; 1.84-fold, 5% confident interval: [1.10–3.06]; Table S3). Together with *ex vivo* enhancer regulation, this suggests that *Dl* is regulated by Mef2 and possibly together with Sd via the *Dl2.6* enhancer.

### Sd and Mef2 activate Dl in SOPs when they are both misexpressed

**Figure 7:**
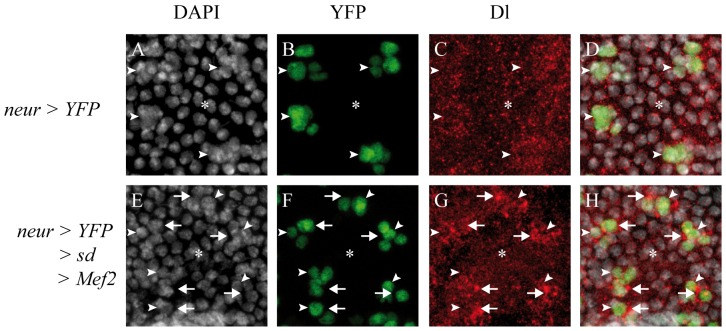
Activation of Dl by Sd/Mef2 in 28 h APF pupa SOPs. In SOPs, visualized with YFP (green) expressed under the control of the *neur-Gal4* driver (arrowheads in A–D), Dl (red) is detectable at the same levels as in non-SOP cells (asterisks in A–D) of the notum. When YFP, *sd* and *Mef2* are overexpressed in SOPs using the same driver (E–H), Dl is up-regulated in SOPs (arrows in G) relative to non-SOP cells (asterisks in G) of the notum. DAPI labeling is shown in gray (A,E). Overlays are shown in D and H.

Our *ex-vivo* data show that Sd and Mef2 synergize to activate the *Dl2.6* enhancer in S2 cells and that Sd and Mef2 overexpression in Dmd8 cells induces a increase in *Dl* mRNA levels. Therefore, to test whether Sd and Mef2 could induce *Dl* overexpression *in vivo*, we overexpressed Sd and Mef2 in SOPs using the *neur-Gal4* driver (Fig. 7). In controls (*neur-Gal4; UAS-H2B::YFP*), SOP clusters express H2B::YFP (Fig. 7B, arrowheads) and Dl immunoreactivity is homogeneously present at low levels in SOPs (Fig. 7C, arrowheads) and non-SOP cells (Fig. 7C, asterisk). When Sd and Mef2 are ectopically misexpressed in SOPs (*neur-Gal4, UAS-H2B::YFP, UAS-sd, UAS-Mef2* genetic background; Fig. 7E-H), Dl expression is increased in SOP clusters (Fig. 7G, arrows; SOP clusters are indicated by arrowheads). Therefore, we conclude that Sd and Mef2 can activate *Dl* in SOPs when they are together misexpressed.

### Mef2 is required for Dl expression in developing DLMs

**Figure 8:**
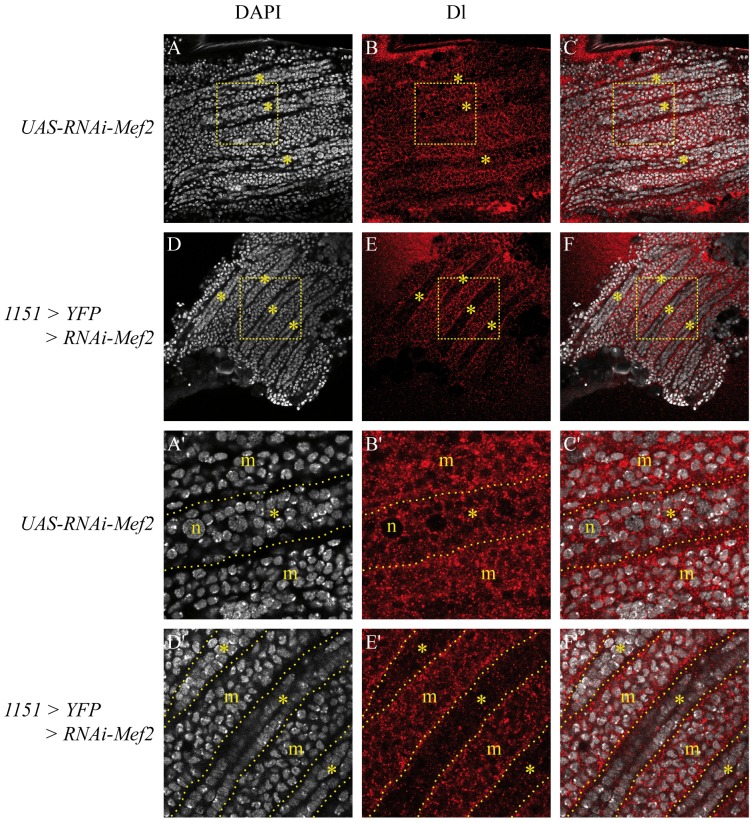
Expression of Dl in IFMs of 24 h APF pupae. Expression of Dl was detected with a anti-Dl antibody in wild-type B, B′–C,C') and *1151-Gal4, UAS-RNAiMef2* (E, E′–F,F') 21 h APF pupae. A'–F' show magnified areas indicated by the dotted squares in A–F respectively. Muscles are visualized with DAPI staining by the specific arrangement of their nuclei (asterisks in A, D, A′, D′; muscles are located between the two dotted lines in A′ and D′). Large nucleus (n in A′) is a larval template nucleus. Myoblasts are located between muscles (m in A′, D′). In wild type pupae, Dl is detected in myoblasts and developing fibers (B, B′; overlay in C, C′). Expression levels in myoblasts and fibers are close (B′). Overexpression of a *RNAi-Mef2* construct in myoblasts and fibers using the *1151-Gal4* driver (D–F, D′–F′) lowers Dl expression in developing fibers compared to myoblasts (E′, compare m to asterisks) and in *1151-Gal4*, *UAS-RNAiMef2* muscles compared to wild type muscles (compare E′, asterisks to B′, asterisk).

We showed that Mef2 is required for *Dl2*.6 activation in developing fibers (Fig. 5) and that Mef2 can activate *Dl* in cultured cells (Fig. 6) and in SOPs when misexepressed with Sd (Fig. 7). To test whether Mef2 is required for Dl expression in 24 h APF developing fibers, we decided to knock down *Mef2* expression using RNA interference. We drove *UAS-RNAi-Mef2* transgene with *1151-Gal4* which is expressed in myoblasts and developing fibers [9]. In 24 h APF pupae, developing fibers can be detected using a DAPI staining: they exhibit a specific alignment of the nuclei (Fig. 8A, D, asterisks, magnification in 8A′, D′, asterisks). Also, occasional large nuclei corresponding to the larval template nuclei can be observed (Fig. 8A′, n). Cells between the developing fibers correspond to myoblasts (Fig. 8A′, F′, m). In wild-type pupae, Dl is expressed in fibers (Fig. 8B, B′, asterisks) and in surrounding myoblasts (Fig. 8B, B′, m). In a previous publication, we detected Dl only in developing fibers [31]. However, we think that this difference is due to technical issues. Indeed, Dl expression has been recently observed in myoblasts [72] and our Dl labeling is very similar to Dl labeling in epithelial cells [73]. In *1151-Gal4, UAS-RNAi-Mef2* genetic context (Fig. 8D–F, magnification in 8D′–F′), Dl expression in myoblasts seems close to Dl expression in controls (Fig. 8E′, m compared to 8B′, m). In contrast, it is clearly reduced in developing fibers (Fig. 8E, asterisks compared to control 8B, asterisks; Fig. 8E′, asterisks, compared to control 8B′, asterisks). Therefore, we concluded that Mef2 is required for Dl expression in developing fibers, but not in the swarming myoblasts.

## Discussion

In this paper, we explored the relationship between the Mef2 myogenic factor and the Notch pathway during muscle fiber development. Previous studies have shown that the Notch pathway activation is required in swarming myoblasts i) to express the anti-differentiation factor Twi [9] and ii) together with Twi, to activate specific targets, such as *Holes in muscle* (Him, [17]). Moreover, it has been shown that persistent activation of the Notch pathway in developing muscles induces muscle degeneration [9]. Together, these data show that the Notch pathway is needed in myoblasts but must be repressed in developing fibers. In myoblasts, Notch interacts with Mef2: it cooperates with Twi to activate *Him* [17] which is a repressor of Mef2 activity [18], [19]. Moreover, muscle degenerations observed when Notch is ectopically activated in fibers is due to persistent activation of *Him* and inhibition of Mef2 activity [19]. Thus, the Notch pathway is activated and Mef2 activity is repressed in undifferentiated myoblasts whereas the Notch pathway is repressed and Mef2 is active in differentiating fibers. To understand how the Notch pathway is repressed in developing fibers we looked for factors active in fibers but not in myoblasts that could be responsible for Notch repression specifically in fibers. We focused on Mef2 as a good candidate because i) increase of Mef2 activity is thought to trigger differentiation since Mef2 overexpression induces early differentiation [15], ii) Mef2 activity is finely tuned during differentiation either through post-transcriptional or post-translational mechanisms [18], , and iii) target gene activation depends on Mef2 activity levels [16].

### Mef2, likely together with Sd, inhibits the Notch pathway

To investigate Mef2 capacity to inhibit the Notch pathway, we ectopically expressed Mef2 in two non-myogenic contexts. Indeed, it has been shown that Mef2 overexpression induces early muscle differentiation [15] and thus Notch repression could be due to the differentiation context rather than to Mef2 expression. Our experiments in the wing disc showed that Mef2 overexpression inhibits activation of Notch known targets at the wing margin (*ct*, *vg*, *wg*; Fig. 1, Figure S1). However, when Mef2 was ectopically expressed in SOPs, we did not observe classical Notch loss of function phenotypes (duplication of the shaft, absence of external cells, [75]). A strong Notch-like phenotype, with no external cells, was only observed when Mef2 and Sd were co-overexpressed (Fig. 2). Moreover, we show that Sd and Mef2 overexpression inhibits the Notch pathway during the first asymmetric division of SOPs (Fig. 3). It seems therefore that inhibition of the Notch pathway by Mef2 in SOPs requires Sd. Differences between these results is likely due to differences in *sd* endogenous expression: whereas *sd* is expressed in the wing pouch [63], we did not detect any *sd* expression in SOPs (data not shown). Thus, it is likely that in the wing pouch, ectopic Mef2 interacts with endogenous Sd and therefore Sd is always required together with Mef2 to inhibit the Notch pathway. This result is consistent with previous studies: Mef2 and Sd can interact physically in *Drosophila* [38], Mef2 and Sd are required for IFM differentiation [41], [76] and Mef2 and Sd cooperate to activate a differentiation specific enhancer in IFMs [41]. Moreover, it has been shown that mammalian orthologs of *sd* and *Mef2* can activate muscle-specific promoters [43].

### Mef2 activates *Dl* through a specific enhancer

We next wanted to determine how the Notch pathway is inhibited by Mef2 and possibly Sd. Since Mef2 and Sd are transcription factors, we hypothesized that they inhibit the pathway by regulating target genes. A previous study isolated 670 cis-regulatory modules (CRMs) bound by Mef2 during embryogenesis [67]. We therefore adopted a bioinformatics approach to rescreen these 670 CRMs for clusters of Sd/Mef2 binding sites, postulating that Sd/Mef2 targets during embryonic myogenesis are also Sd/Mef2 targets during IFM development. We isolated a 2.6 kb sequence, *Dl2.6*, which was predicted to be a *Dl* enhancer (see supplementary materials in [67]). Using a transgenic approach, we showed that *Dl2.6* is activated in IFMs between 16 h and 36 h APF (Fig. 4) where Sd and Mef2 activate the *vestigial* adult muscle enhancer [41].

We show that Mef2 can activate *Dl2.6* in myogenic and non-myogenic cultured cells and that *Mef2 in vivo* knock-down induces a decrease in *Dl2.6* activation as well as Dl expression in muscle fibers (Fig. 5, 8). Interestingly, when *Mef2* is down-regulated in the entire IFM myogenic lineage, a significant Dl decrease is observed in developing IFMs whereas Dl expression in myolasts is not significantly changed (myoblasts and fibers; Fig. 8). This shows that Mef2 is required for *Dl* expression only in developing IFMs, likely through the activation of the *Dl2.6* enhancer. We tried to test whether Sd is involved in *Dl2.6* activation and *Dl* expression. *In vivo* experiments did not allow us to conclude on this question. Indeed in *sd^11L^* and *sd^68L^* mutants, *Dl2.6* is activated. However, these alleles are not as early lethal as *sd* null alleles and are likely to retain a part of Sd activity [49]. Therefore we cannot exclude that this remnant activity is sufficient to activate the *Dl2.6* enhancer. *Ex vivo* experiments seemed to be more conclusive: Sd and Mef2 synergize to activate *Dl2.6* in S2 cells (Fig. 6A). On the other hand an inhibitory effect of Sd is observed in Dmd8 cells (Fig. 6B). This inhibition may be due to a squelching effect [71]. Such phenomenon has been previously described with the Sd/Vg transcription factor. Indeed, the SRF-A enhancer is activated by Sd and Vg, but activation by Sd/Vg diminished with increasing Sd concentrations [34]. This is interpreted as a titration of the Vg factor by Sd: when Sd is in excess, Sd-Vg dimers are replaced on promoters by Sd monomers. In the same way, an excess of Sd could titrate the Mef2 factor and therefore diminish the activation of the *Dl2.6* enhancer. Thus, considering i) cultured cell results, ii) that Sd-Mef2 ectopic expression in SOPs induces Dl and iii) that Sd-Mef2 overexpression in Dmd8 cells induces an increase in *Dl* mRNA levels, we favored the hypothesis that Sd is also involved in *Dl2.6* activation and *Dl* expression. However, further studies are needed to conclude on this point.

### Role of Mef2 and *Dl* in Notch inhibition

Together these data show that Mef2 interacts with the Notch pathway during IFM differentiation, at least by activating *Dl*. However, if *Dl* can participate in mechanisms involved in Notch inhibition, it is not the only factor responsible for it. Indeed, *Dl* is expressed in myoblasts (Fig. 8) were the Notch pathway is activated [9], suggesting that *Dl* activation by Mef2 in developing fibers is a mechanism for maintaining *Dl* expression in the myogenic lineage. Moreover, *Dl* expression is not sufficient to inactivate the pathway: *Dl* over-activation in SOPs or myoblasts using the *neur-Gal4* or *1151-Gal4* drivers respectively failed to reproduce *Mef2* overexpression or Notch phenotypes (data not shown). This last observation is similar to previously published results of *Dl* overexpression in SOPs [77].

In a previous study, we have shown that *vg* is also required for Notch inhibition in developing DLMs and thus for a proper muscle differentiation. We showed that in absence of *vg*, *fng* is not expressed in DLMs and that forcing *fng* expression in the *vg* mutant is sufficient to rescue the muscle phenotype [31]. *fng* encodes a glycosyltransferase that modifies the Notch receptor, increasing its affinity for its ligand Dl [22]–[24]. In wild-type flies, it is expressed in developing fibers but not in myoblasts [31]. It seemed therefore that the modifications of Notch by Fng, and thus the modification of Notch affinity for Dl, is a critical step in Notch repression in developing DLMs and therefore for DLM differentiation. Here we show that Mef2 can inhibit the Notch pathway and that Mef2 is responsible for Dl expression in developing fibers. Therefore, Notch inhibition is associated with *fng* and *Dl* expression in developing fibers. These results may appear contradictory as Dl activates the Notch pathway. However, Dl is expressed in the signal sending cell. Moreover, in the wing disc, the Notch pathway is inactive in cells expressing Notch and Dl. Indeed, a mechanism, called cis-inhibition, involving the Notch receptor and its ligands in the repression of the pathway has been previously described (for review, see [78]). In the wing pouch of the wing imaginal disc, Notch is ubiquitously expressed, *Dl* is only expressed in the ventral part and *fng* is only expressed in the dorsal part. When *Dl* overexpressing clones are induced in the dorsal part, the Notch pathway is activated at the boundary of the clones, but not in the clonal cells where *Dl* and *fng* are expressed [79]. A similar result is observed when *fng* overexpressing clones are induced in the ventral part [80]. Thus, in cells expressing *N*, *Dl* and *fng*, the Notch pathway is inactivated. Therefore, activation of *Dl* by Mef2 in developing fibers, where *fng* is expressed, could be necessary for Notch inactivation through a cis-inhibition mechanism. Interestingly, *fng* is also expressed in the wing disc [81] and SOPs (data not shown) where Mef2 ectopic expression inhibits the Notch pathway.

Since *fng* is required for Notch inhibition and Mef2 inhibits the Notch pathway, we also asked whether Mef2 can activate *fng* as well. However, using the *fng^35UZ^-LacZ* transgene as a reporter, we never observed any activation of *fng* by Mef2. Moreover, we did not find Sd/Mef2 binding site clusters in and around the *fng* gene. However, we showed that Sd and Mef2 can activate a muscle specific enhancer of *vg* [41] and that Vg is required for *fng* expression [31]. Therefore, even if *fng* is not directly activated by Sd/Mef2, it is possible that Sd and Mef2 may be involved in *fng* activation through *vg* activation.

### Triggering differentiation: a switch in Mef2-Notch interactions?

The Notch pathway is active in myoblasts [9] where it activates *Him*, a repressor of Mef2 transcriptional activity [18], [19]. In contrast, in developing IFMs, Notch is not active [9], *Him* is not expressed [19] and therefore Mef2 is active [76]. It has been shown that Mef2 activates different target genes according to its activity level [16], [45] and that Mef2 overexpression in myoblasts induces early differentiation [15]. Soler et al. proposed that a balance of Him and Mef2 activities could regulate muscle differentiation: high levels of Him repress Mef2 activity and consequently differentiation, while high levels of Mef2 overwhelm repression by Him and induce muscle differentiation [19]. Here we propose that one effect of Mef2 high activity levels could be to trigger the repression of the Notch pathway, switching from a stable undifferentiated state in which the Notch pathway represses Mef2 activity through Him (Figure S1, A) to a stable differentiating state in which Mef2 activity remains high through a positive feedback loop and represses the Notch pathway (Figure S1, B). This inactivation could occur through a cis-inhibition mechanism, since Mef2 activates *Dl* in developing fibers.

## Supporting Information

Figure S1 A: in AMPs, the Notch pathway activates *twi* (1) that inhibits muscle differentiation (2). The pro-differentiation gene *Mef2* is activated by Twi (4) but the transcriptional activity of Mef2 is repressed by Him (5), a target of Twi and Notch (3). B: in differentiating fibers, Notch is not active and therefore Him and Twi are absent. Mef2, for which levels increase due to a positive feedback, is transcriptionaly active and triggers muscle differentiation.
(TIF)

Figure S2 **Notch repression by Mef2 in the wing disc.** Panels B and H show Vg and Wg expression patterns in wild type third instar larva wing disc (DAPI in A, G). When Mef2 is overexpressed along the AP boundary of the third instar larva wing disc using the *ptc-Gal4* driver (D, J), neither Vg (E, arrowhead) or Wg (K, arrowheads), two known targets of the Notch pathway, are detected where Mef2 is ectopically expressed (overlay in F, L, DAPI in C, I).
(TIF)

Figure S3 **Schematic representation of SOP development.** Notch pathway activity is represented by red outlining. *Prospero* expression is represented in blue. (A) In normal developmental circumstances, SOP microchaete development starts by specification of the pI cell by lateral inhibition. At 17 h APF, this cell will undergo an asymmetrical division to give rise to the pIIa and pIIb cells. The pIIa will give rise to the external shaft and socket cells. The pIIb cell will divide asymmetrically to give rise to the pIIIb cell and a glial cell that will degenerate. The pIIIb cell gives rise to the internal cells of the sensory organ, the shaft cell and the neuron. *Prospero* staining at 21 h APF (purple rectangle) reveals one *prospero* positive cell in which the Notch pathway is not active. (B) Theoretically, Notch pathway repression after pI specification and throughout SOP development should induce two pIIb cells after pI division and only glial cells after pIIb division.
(TIF)

Figure S4 **Schematic representation of the** ***Dl2.6*** **enhancer (3R: 15,142,950..15,145,589).** Predicted Mef2 binding sites are shown as yellow arrowheads. Predicted Sd binding sites are shown as gray arrowheads.
(TIF)

Figure S5 ***Dl2.6*** **activation in** ***sd^11L^*** **and** ***sd^68L^*** **mutants.** In 21 h APF wild-type male pupae (*FM7*/Y; *Dl2.6-GFP*/+), the *Dl2.6* enhancer is activated in developing IFMs (B, DAPI in A, overlay in C). In 21 h APF *sd^11L^* and *sd^68L^* male pupae (respectively *FM7*/*sd^11L^*; *Dl2.6-GFP*/+ and *FM7*/*sd^68L^*; *Dl2.6-GFP*/+), the *Dl2.6* enhancer is also activated (respectively E and H; DAPI in D, G; overlays in F, I).
(TIF)

Table S1 **Raw data for Dl2.6 activity in S2 cells.**
(XLS)

Table S2 **Raw data for Dl2.6 activity in Dmd8 cells.**
(XLSX)

Table S3 **Q-RT-PCR were performed on cells transfected by an empty pCasPer vector or on cells transfected by** ***pCasPer-hsp70-Mef2*** **and** ***pCasPer-hsp70-sd*** **vectors.** Each point was repeated 3 times (see material and methods; Casper1-3, SDMEF1-3). dCp (Cp RP49 - Cp Dl) was calculated to normalize data according to the RP49 housekeeping gene expression. The mean^(1)^ and the confidence interval^(2)^ were calculated in each condition. ddCp^(3)^ was obtained substracting dCp (SDMEF condition) from dCp (Casper condition). Confidence interval^(4)^ is calculated adding or removing the confidence interval in ^(2)^. Increase ^(5)^ and the confidence interval ^(6)^ are obtained by the formula: Increase = 2^ddCp^. (Pfaffl, 2001).
(XLSX)

## References

[1] Abmayr SM, Pavlath GK (2012) Myoblast fusion: lessons from flies and mice. Development 139: 641–656. 22274696 10.1242/dev.068353 PMC3265056

[2] Taylor MV (2006) Comparison of muscle development in *Drosophila* and vertebrates. In: H Sink, editor editors. Muscle development in *Drosophila*. Georgetown/New-York: Landes Bioscience/Springer. pp. 169–203.

[3] Baylies MK, Bate M, Gomez MR (1998) Myogenesis: A View from *Drosophila* . Cell 93: 921–927. 9635422 10.1016/s0092-8674(00)81198-8

[4] Dutta D, VijayRaghavan K (2006) Metamorphosis and the formation of the adult musculature. In: H Sink, editor editors. Muscle development in *Drosophila*. Georgetown/New-York: Landes Bioscience/Springer. pp. 125–142.

[5] Crossley AC (1978) The morphology and development of the *Drosophila muscular system* In: M Ashburner and T. R. F Wright, editors. The Genetics and biology of *Drosophila*. London: Academic Press. pp. 499–560.

[6] Fernandes J, Bate M, Vijayraghavan K (1991) Development of the indirect flight muscles of *Drosophila* . Development 113: 67–77. 1765009 10.1242/dev.113.1.67

[7] Bate M, Rushton E, Currie DA (1991) Cells with persistent twist expression are the embryonic precursors of adult muscles in *Drosophila* . Development 113: 79–89. 1765010 10.1242/dev.113.1.79

[8] Thisse B, Stoetzel C, Gorostiza-Thisse C, Perrin-Schmitt F (1988) Sequence of the twist gene and nuclear localization of its protein in endomesodermal cells of early *Drosophila* embryos. EMBO J 7: 2175–2183. 3416836 10.1002/j.1460-2075.1988.tb03056.x PMC454534

[9] Anant S, Roy S, VijayRaghavan K (1998) Twist and Notch negatively regulate adult muscle differentiation. Development 125: 1361–1369. 9502718 10.1242/dev.125.8.1361

[10] Cripps RM (1997) The myogenic regulatory gene Mef2 is a direct target for transcriptional activation by Twist during *Drosophila* myogenesis. Genes Dev 12: 422–434. 10.1101/gad.12.3.422 PMC316486 9450935

[11] Bour BA, O'Brien MA, Lockwood WL, Goldstein ES, Bodmer R, et al (1995) *Drosophila* MEF2, a transcription factor that is essential for myogenesis. Genes Dev 9: 730–741. 7729689 10.1101/gad.9.6.730

[12] Lilly B, Zhao B, Ranganayakulu G, Paterson BM, Schulz RA, et al (1995) Requirement of MADS domain transcription factor D-MEF2 for muscle formation in *Drosophila* . Science 267: 688–693. 7839146 10.1126/science.7839146

[13] Cripps RM, Olson EN (1998) Twist Is Required for Muscle Template Splitting during Adult *Drosophila* Myogenesis. Dev Biol 203: 106–115. 9806776 10.1006/dbio.1998.9040

[14] Nguyen T, Wang J, Schulz RA (2002) Mutations within the conserved MADS box of the D-MEF2 muscle differentiation factor result in a loss of DNA binding ability and lethality in *Drosophila* . Differentiation 70: 438–446. 12366381 10.1046/j.1432-0436.2002.700806.x

[15] Lovato TL, Benjamin AR, Cripps RM (2005) Transcription of *Myocyte enhancer factor-2* in adult *Drosophila* myoblasts is induced by the steroid hormone ecdysone. Dev Biol 288: 612–621. 16325168 10.1016/j.ydbio.2005.09.007

[16] Elgar SJ, Han J, Taylor MV (2008) mef2 activity levels differentially affect gene expression during *Drosophila* muscle development. Proc Natl Acad Sci U S A 105: 918–923. 18198273 10.1073/pnas.0711255105 PMC2242723

[17] Bernard F, Krejci A, Housden B, Adryan B, Bray SJ (2010) Specificity of Notch pathway activation: Twist controls the transcriptional output in adult muscle progenitors. Development 137: 2633–2642. 20610485 10.1242/dev.053181 PMC2910383

[18] Liotta D, Han J, Elgar S, Garvey C, Han Z, et al (2007) The Him Gene Reveals a Balance of Inputs Controlling Muscle Differentiation in *Drosophila* . Curr Biol 17: 1409–1413. 17702578 10.1016/j.cub.2007.07.039 PMC1955682

[19] Soler C, Taylor MV (2009) The Him gene inhibits the development of *Drosophila* flight muscles during metamorphosis. Mech Dev 126: 595–603. 19324085 10.1016/j.mod.2009.03.003

[20] Andersson ER, Sandberg R, Lendahl U (2011) Notch signaling: simplicity in design, versatility in function. Development 138: 3593–3612. 21828089 10.1242/dev.063610

[21] Bray SJ (2006) Notch signalling: a simple pathway becomes complex. Nat Rev Mol Cell Biol 7: 678–689. 16921404 10.1038/nrm2009

[22] Moloney DJ, Panin VM, Johnston SH, Chen J, Shao L, et al (2000) Fringe is a glycosyltransferase that modifies Notch. Nature 406: 369–375. 10935626 10.1038/35019000

[23] Panin VM, Papayannopoulos V, Wilson R, Irvine KD (1997) Fringe modulates Notch-ligand interactions. Nature 387: 908–912. 9202123 10.1038/43191

[24] Bruckner K, Perez L, Clausen H, Cohen S (2000) Glycosyltransferase activity of Fringe modulates Notch-Delta interactions. Nature 406: 411–415. 10935637 10.1038/35019075

[25] Artavanis-Tsakonas S, Simpson P (1991) Choosing a cell fate: a view from the Notch locus. Trends Genet 7: 403–408. 1668193 10.1016/0168-9525(91)90264-q

[26] Artavanis-Tsakonas S (1995) Notch signaling. Science 268: 225–232. 7716513 10.1126/science.7716513

[27] Bray S (1998) Notch signalling in *Drosophila*: three ways to use a pathway. Semin Cell Dev Biol 9: 591–597. 10075488 10.1006/scdb.1998.0262

[28] Artavanis-Tsakonas (1999) Notch signaling: cell fate control and signal integration in development. Science 284: 770–776. 10221902 10.1126/science.284.5415.770

[29] Lai EC (2004) Notch signaling: control of cell communication and cell fate. Development 131: 965–973. 14973298 10.1242/dev.01074

[30] Bray S (2010) Notch Targets and Their Regulation. Curr Top Dev Biol. Academic Press. pp. 253–275. 10.1016/S0070-2153(10)92008-5 20816398

[31] Bernard F, Dutriaux A, Silber J, Lalouette A (2006) Notch pathway repression by vestigial is required to promote indirect flight muscle differentiation in *Drosophila melanogaster* . Dev Biol 295: 164–177. 16643882 10.1016/j.ydbio.2006.03.022

[32] Williams JA, Bell JB, Carroll SB (1991) Control of *Drosophila* wing and haltere development by the nuclear vestigial gene product. Genes Dev 5: 2481–2495. 1752439 10.1101/gad.5.12b.2481

[33] Simmonds AJ, Liu X, Soanes KH, Krause HM, Irvine KD, et al (1998) Molecular interactions between Vestigial and Scalloped promote wing formation in *Drosophila* . Genes Dev 12: 3815–3820. 9869635 10.1101/gad.12.24.3815 PMC317270

[34] Halder G, Polaczyk P, Kraus ME, Hudson A, Kim J, et al (1998) The Vestigial and Scalloped proteins act together to directly regulate wing-specific gene expression in *Drosophila* . Genes Dev 12: 3900–3909. 9869643 10.1101/gad.12.24.3900 PMC317267

[35] Vaudin P, Delanoue R, Davidson I, Silber J, Zider A (1999) TONDU (TDU), a novel human protein related to the product of vestigial (vg) gene of *Drosophila melanogaster* interacts with vertebrate TEF factors and substitutes for Vg function in wing formation. Development 126: 4807–4816. 10518497 10.1242/dev.126.21.4807

[36] Klein T, Arias AM (1999) The vestigial gene product provides a molecular context for the interpretation of signals during the development of the wing in *Drosophila* . Development 126: 913–925. 9927593 10.1242/dev.126.5.913

[37] Zider A, Paumard-Rigal S, Frouin I, Silber J (1998) The vestigial gene of *Drosophila melanogaster* is involved in the formation of the peripheral nervous system: genetic interactions with the scute gene. J Neurogenet 12: 87–99. 10197159 10.3109/01677069809167258

[38] Deng H, Hughes SC, Bell JB, Simmonds AJ (2009) Alternative Requirements for Vestigial, Scalloped, and Dmef2 during Muscle Differentiation in *Drosophila melanogaster* . Mol Biol Cell 20: 256–269. 18987343 10.1091/mbc.E08-03-0288 PMC2613084

[39] Bernard F, Lalouette A, Gullaud M, Jeantet A, Cossard R, et al (2003) Control of apterous by vestigial drives indirect flight muscle development in *Drosophila* . Dev Biol 260: 391–403. 12921740 10.1016/s0012-1606(03)00255-0

[40] Sudarsan V, Anant S, Guptan P, VijayRaghavan K, Skaer H (2001) Myoblast Diversification and Ectodermal Signaling in *Drosophila* . Dev Cell 1: 829–839. 11740944 10.1016/s1534-5807(01)00089-2

[41] Bernard F, Kasherov P, Grenetier S, Dutriaux A, Zider A, et al (2009) Integration of differentiation signals during indirect flight muscle formation by a novel enhancer of *Drosophila* vestigial gene. Dev Biol 332: 258–272. 19500564 10.1016/j.ydbio.2009.05.573

[42] Maeda T, Chapman DL, Stewart AF (2002) Mammalian Vestigial-like 2, a Cofactor of TEF-1 and MEF2 Transcription Factors That Promotes Skeletal Muscle Differentiation. J Biol Chem 277: 48889–48898. 12376544 10.1074/jbc.M206858200

[43] Maeda T, Gupta MP, Stewart AF (2002) TEF-1 and MEF2 transcription factors interact to regulate muscle-specific promoters. Biochemical and Biophysical Research Communications 294: 791–797. 12061776 10.1016/S0006-291X(02)00556-9

[44] Ng M, Diaz-Benjumea FJ, Vincent J-P, Wu J, Cohen SM (1996) Specification of the wing by localized expression of wingless protein. Nature 381: 316–318. 8692268 10.1038/381316a0

[45] Gunthorpe D, Beatty KE, Taylor MV (1999) Different Levels, but Not Different Isoforms, of the *Drosophila* Transcription Factor DMEF2 Affect Distinct Aspects of Muscle Differentiation. Dev Biol 215: 130–145. 10525355 10.1006/dbio.1999.9449

[46] Bellaiche Y, Gho M, Kaltschmidt JA, Brand AH, Schweisguth F (2001) Frizzled regulates localization of cell-fate determinants and mitotic spindle rotation during asymmetric cell division. Nat Cell Biol 3: 50–57. 11146626 10.1038/35050558

[47] Dietzl G, Chen D, Schnorrer F, Su K-C, Barinova Y, et al (2007) A genome-wide transgenic RNAi library for conditional gene inactivation in *Drosophila* . Nature 448: 151–156. 17625558 10.1038/nature05954

[48] Kumar JP, Moses K (2001) EGF Receptor and Notch Signaling Act Upstream of Eyeless/Pax6 to Control Eye Specification. Cell 104: 687–697. 11257223 10.1016/s0092-8674(01)00265-3

[49] Srivastava A, Simmonds AJ, Garg A, Fossheim L, Campbell SD, et al (2004) Molecular and functional analysis of scalloped recessive lethal alleles in *Drosophila melanogaster* . Genetics 166: 1833–1843. 15126402 10.1534/genetics.166.4.1833 PMC1470810

[50] Nolo R, Abbott LA, Bellen HJ (2000) Senseless, a Zn Finger Transcription Factor, Is Necessary and Sufficient for Sensory Organ Development in *Drosophila* . Cell 102: 349–362. 10975525 10.1016/s0092-8674(00)00040-4

[51] Goulev Y, Fauny JD, Gonzalez-Marti B, Flagiello D, Silber J, et al (2008) SCALLOPED interacts with YORKIE, the nuclear effector of the hippo tumor-suppressor pathway in *Drosophila* . Curr Biol 18: 435–441. 18313299 10.1016/j.cub.2008.02.034

[52] Barolo S, Carver LA, Posakony JW (2000) GFP and beta-galactosidase transformation vectors for promoter/enhancer analysis in *Drosophila*. Biotechniques 29: 726, 728, 730, 732. 10.2144/00294bm10 11056799

[53] Sambrook J, Fritsch EF, Maniatis T (1989) Molecular cloning: a laboratory manual. New York.

[54] Chintapalli VR, Wang J, Dow JAT (2007) Using FlyAtlas to identify better *Drosophila melanogaster* models of human disease. Nat Genet 39: 715–720. 17534367 10.1038/ng2049

[55] Pfaffl MW (2001) A new mathematical model for relative quantification in real-time RT-PCR. Nucleic Acids Res 29: e45. 11328886 10.1093/nar/29.9.e45 PMC55695

[56] Lovato TL, Adams MM, Baker PW, Cripps RM (2009) A Molecular Mechanism of Temperature Sensitivity for Mutations Affecting the *Drosophila* Muscle Regulator Myocyte Enhancer Factor-2. Genetics 183: 107–117. 19564485 10.1534/genetics.109.105056 PMC2746136

[57] Olson EN, Perry M, Schulz RA (1995) Regulation of Muscle Differentiation by the MEF2 Family of MADS Box Transcription Factors. Dev Biol 172: 2–14. 7589800 10.1006/dbio.1995.0002

[58] Potthoff MJ, Olson EN (2007) MEF2: a central regulator of diverse developmental programs. Development 134: 4131–4140. 17959722 10.1242/dev.008367

[59] Soler C, Han J, Taylor MV (2012) The conserved transcription factor Mef2 has multiple roles in adult *Drosophila* musculature formation. Development 139: 1270–1275. 22357930 10.1242/dev.077875

[60] Taylor MV (1995) Muscle Development: Making *Drosophila* muscle. Curr Biol 5: 740–742. 7583119 10.1016/s0960-9822(95)00149-7

[61] Lai EC, Bodner R, Posakony JW (2000) The enhancer of split complex of *Drosophila* includes four Notch-regulated members of the bearded gene family. Development 127: 3441–3455. 10903170 10.1242/dev.127.16.3441

[62] de Celis JF, Garcia-Bellido A, Bray SJ (1996) Activation and function of Notch at the dorsal-ventral boundary of the wing imaginal disc. Development 122: 359–369. 8565848 10.1242/dev.122.1.359

[63] Campbell S, Inamdar M, Rodrigues V, Raghavan V, Palazzolo M, et al (1992) The scalloped gene encodes a novel, evolutionarily conserved transcription factor required for sensory organ differentiation in *Drosophila* . Genes Dev 6: 367–379. 1547938 10.1101/gad.6.3.367

[64] Campos-Ortega JA (1988) Cellular interactions during early neurogenesis of *Drosophila melanogaster* . Trends Neurosci 11: 400–405. 2469206 10.1016/0166-2236(88)90077-x

[65] Manning L, Doe CQ (1999) Prospero distinguishes sibling cell fate without asymmetric localization in the *Drosophila* adult external sense organ lineage. Development 126: 2063–2071. 10207132 10.1242/dev.126.10.2063

[66] Reddy GV, Rodrigues V (1999) Sibling cell fate in the *Drosophila* adult external sense organ lineage is specified by prospero function, which is regulated by Numb and Notch. Development 126: 2083–2092. 10207134 10.1242/dev.126.10.2083

[67] Sandmann T, Jensen LJ, Jakobsen JS, Karzynski MM, Eichenlaub MP, et al (2006) A Temporal Map of Transcription Factor Activity: Mef2 Directly Regulates Target Genes at All Stages of Muscle Development. Dev Cell 10: 797–807. 16740481 10.1016/j.devcel.2006.04.009

[68] Schneider I (1972) Cell lines derived from late embryonic stages of *Drosophila melanogaster* . J Embryol Exp Morphol 27: 353–365. 4625067

[69] Krejci A, Bernard F, Housden BE, Collins S, Bray SJ (2009) Direct Response to Notch Activation: Signaling Crosstalk and Incoherent Logic. Sci Signal 2: ra1. 19176515 10.1126/scisignal.2000140

[70] Ui K, Ueda R, Miyake T (1987) Cell lines from imaginal discs of *Drosophila melanogaster* . In Vitro Cell Dev Biol 23: 707–711. 3117765 10.1007/BF02620984

[71] Cahill MA, Ernst WH, Janknecht R, Nordheim A (1994) Regulatory squelching. FEBS Lett 344: 105–108. 8187867 10.1016/0014-5793(94)00320-3

[72] Gildor B, Schejter ED, Shilo BZ (2012) Bidirectional Notch activation represses fusion competence in swarming adult *Drosophila* myoblasts. Development 139: 4040–4050. 23048185 10.1242/dev.077495

[73] Benhra N, Vignaux F, Dussert A, Schweisguth F, Le Borgne R (2010) Neuralized promotes basal to apical transcytosis of delta in epithelial cells. Mol Biol Cell 21: 2078–2086. 20410139 10.1091/mbc.E09-11-0926 PMC2883951

[74] Chen Z, Liang S, Zhao Y, Han Z (2012) miR-92b regulates Mef2 levels through a negative-feedback circuit during *Drosophila* muscle development. Development 139: 3543–3552. 22899845 10.1242/dev.082719 PMC3436111

[75] Le Bras S, Rondanino C, Kriegel-Taki G, Dussert A, Le Borgne R (2012) Genetic identification of intracellular trafficking regulators involved in Notch-dependent binary cell fate acquisition following asymmetric cell division. J Cell Sci 125: 4886–4901. 22825875 10.1242/jcs.110171

[76] Ranganayakulu G, Zhao B, Dokidis A, Molkentin JD, Olson EN, et al (1995) A Series of Mutations in the D-MEF2 Transcription Factor Reveal Multiple Functions in Larval and Adult Myogenesis in *Drosophila* . Dev Biol 171: 169–181. 7556894 10.1006/dbio.1995.1269

[77] Doherty D, Jan LY, Jan YN (1997) The *Drosophila* neurogenic gene big brain, which encodes a membrane-associated protein, acts cell autonomously and can act synergistically with Notch and Delta. Development 124: 3881–3893. 9367444 10.1242/dev.124.19.3881

[78] del Alamo D, Rouault H, Schweisguth F (2011) Mechanism and Significance of cis-Inhibition in Notch Signalling. Curr Biol 21: R40–R47. 21215938 10.1016/j.cub.2010.10.034

[79] de Celis JF, Bray S (1997) Feed-back mechanisms affecting Notch activation at the dorsoventral boundary in the *Drosophila* wing. Development 124: 3241–3251. 9310319 10.1242/dev.124.17.3241

[80] de Celis JF, Bray SJ (2000) The Abruptex domain of Notch regulates negative interactions between Notch, its ligands and Fringe. Development 127: 1291–1302. 10683181 10.1242/dev.127.6.1291

[81] Irvine KD, Wieschaus E (1994) fringe, a boundary-specific signaling molecule, mediates interactions between dorsal and ventral cells during *Drosophila* wing development. Cell 79: 595–606. 7954826 10.1016/0092-8674(94)90545-2

