# Supplementary figures and images for: Mef2 Interacts with the Notch Pathway during Adult Muscle Development in Drosophila melanogaster
Source: PLoS One. 2014 Sep 23;9(9):e108149. doi: 10.1371/journal.pone.0108149 (PMC4172597; doi:10.1371/journal.pone.0108149)

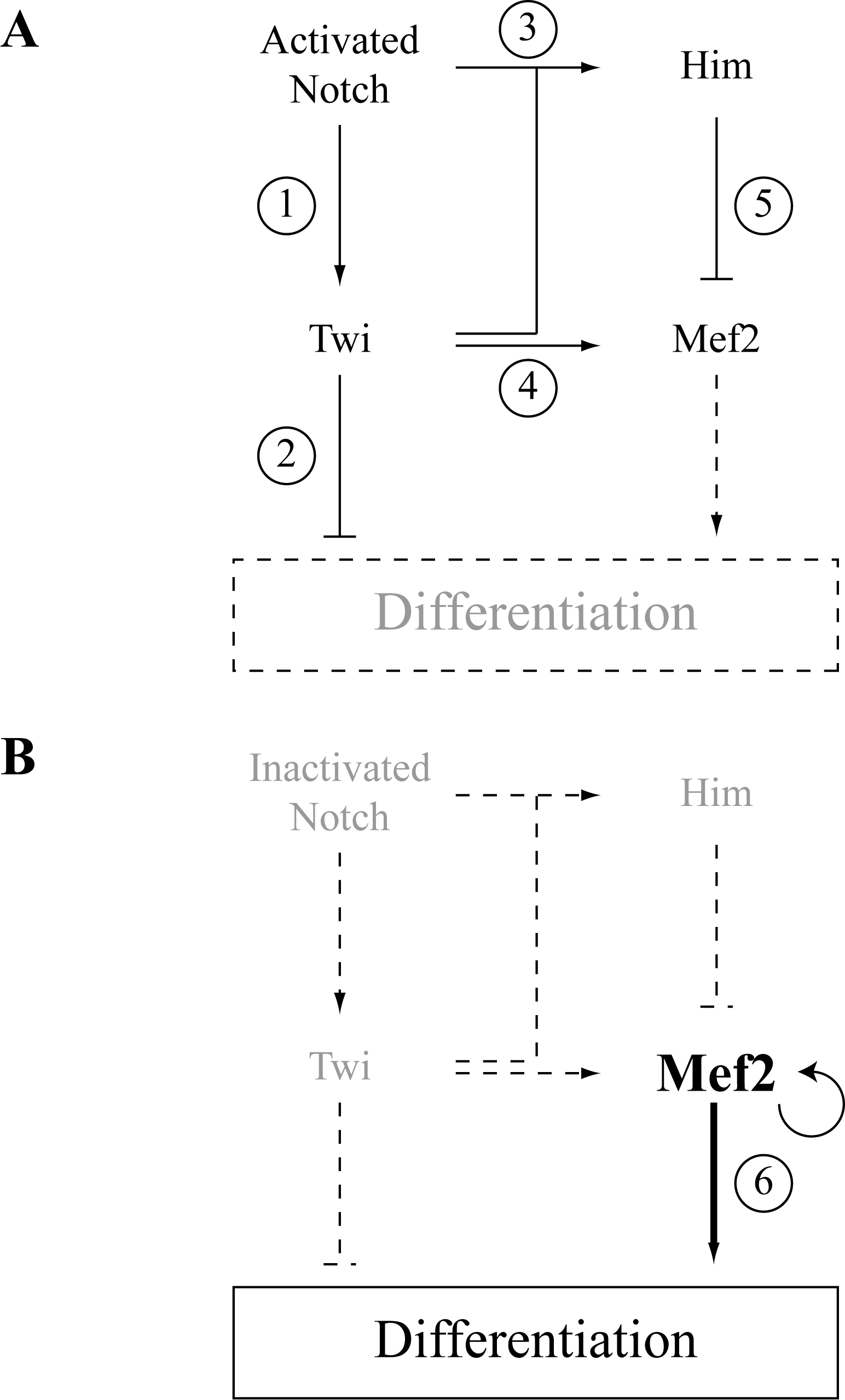

Supplement: Figure S1 — A: in AMPs, the Notch pathway activates twi (1) that inhibits muscle differentiation (2). The pro-differentiation gene Mef2 is activated by Twi (4) but the transcriptional activity of Mef2 is repressed by Him (5), a target of Twi and Notch (3). B: in differentiating fibers, Notch is not active and therefore Him and Twi are absent. Mef2, for which levels increase due to a positive feedback, is transcriptionaly active and triggers muscle differentiation. (TIF) [file pone.0108149.s001.tif]

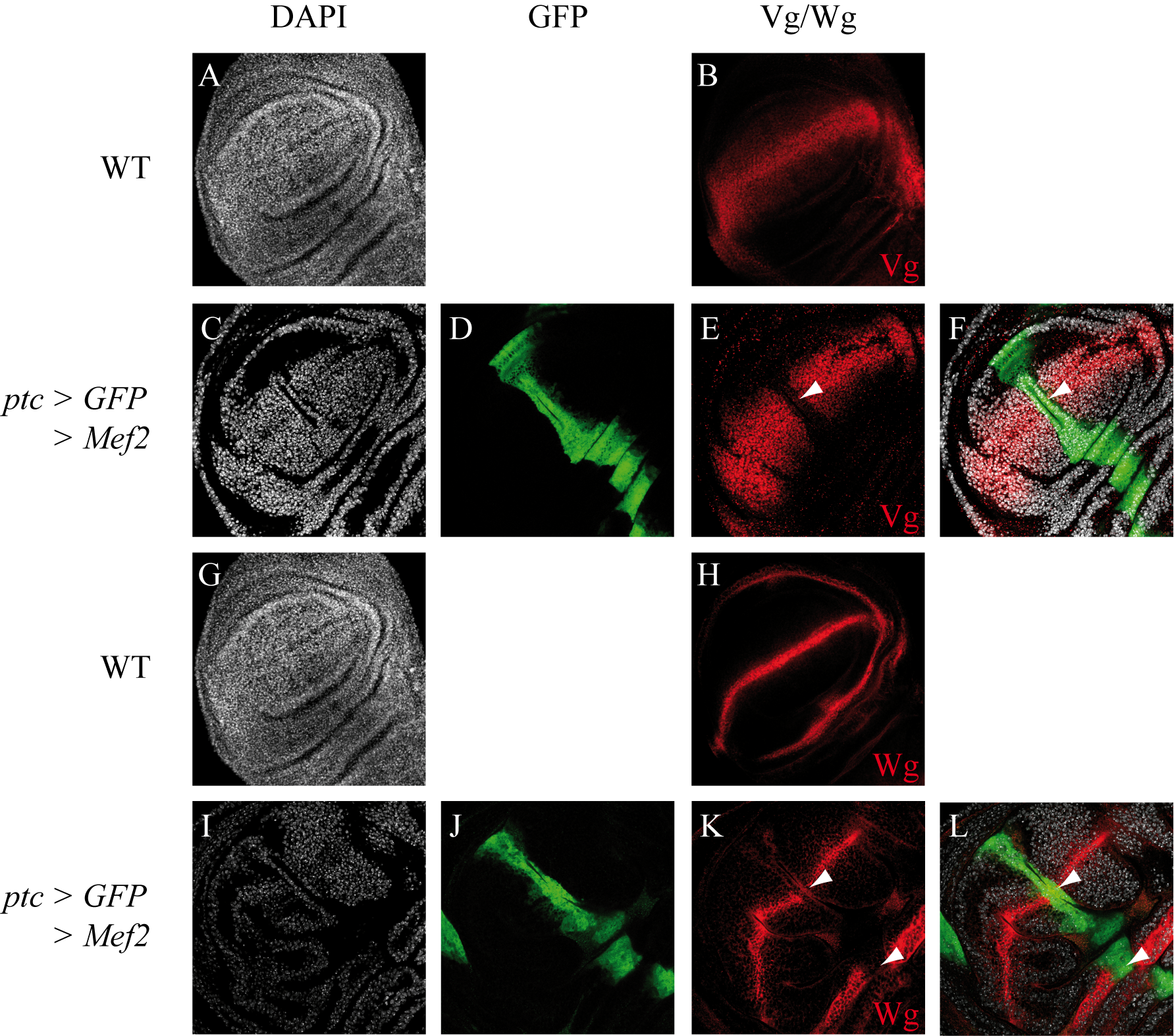

Supplement: Figure S2 — Notch repression by Mef2 in the wing disc. Panels B and H show Vg and Wg expression patterns in wild type third instar larva wing disc (DAPI in A, G). When Mef2 is overexpressed along the AP boundary of the third instar larva wing disc using the ptc-Gal4 driver (D, J), neither Vg (E, arrowhead) or Wg (K, arrowheads), two known targets of the Notch pathway, are detected where Mef2 is ectopically expressed (overlay in F, L, DAPI in C, I). (TIF) [file pone.0108149.s002.tif]

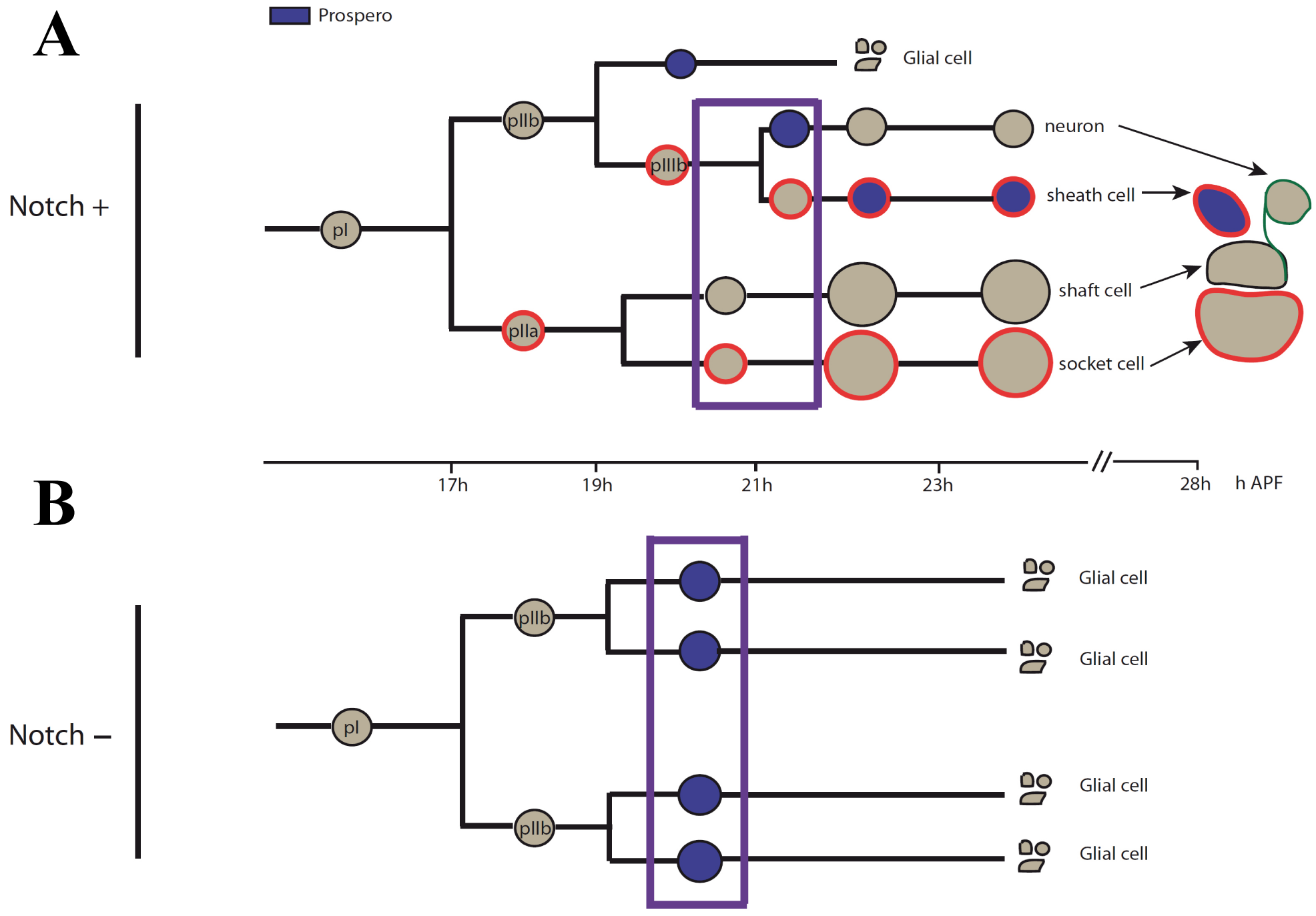

Supplement: Figure S3 — Schematic representation of SOP development. Notch pathway activity is represented by red outlining. Prospero expression is represented in blue. (A) In normal developmental circumstances, SOP microchaete development starts by specification of the pI cell by lateral inhibition. At 17 h APF, this cell will undergo an asymmetrical division to give rise to the pIIa and pIIb cells. The pIIa will give rise to the external shaft and socket cells. The pIIb cell will divide asymmetrically to give rise to the pIIIb cell and a glial cell that will degenerate. The pIIIb cell gives rise to the internal cells of the sensory organ, the shaft cell and the neuron. Prospero staining at 21 h APF (purple rectangle) reveals one prospero positive cell in which the Notch pathway is not active. (B) Theoretically, Notch pathway repression after pI specification and throughout SOP development should induce two pIIb cells after pI division and only glial cells after pIIb division. (TIF) [file pone.0108149.s003.tif]

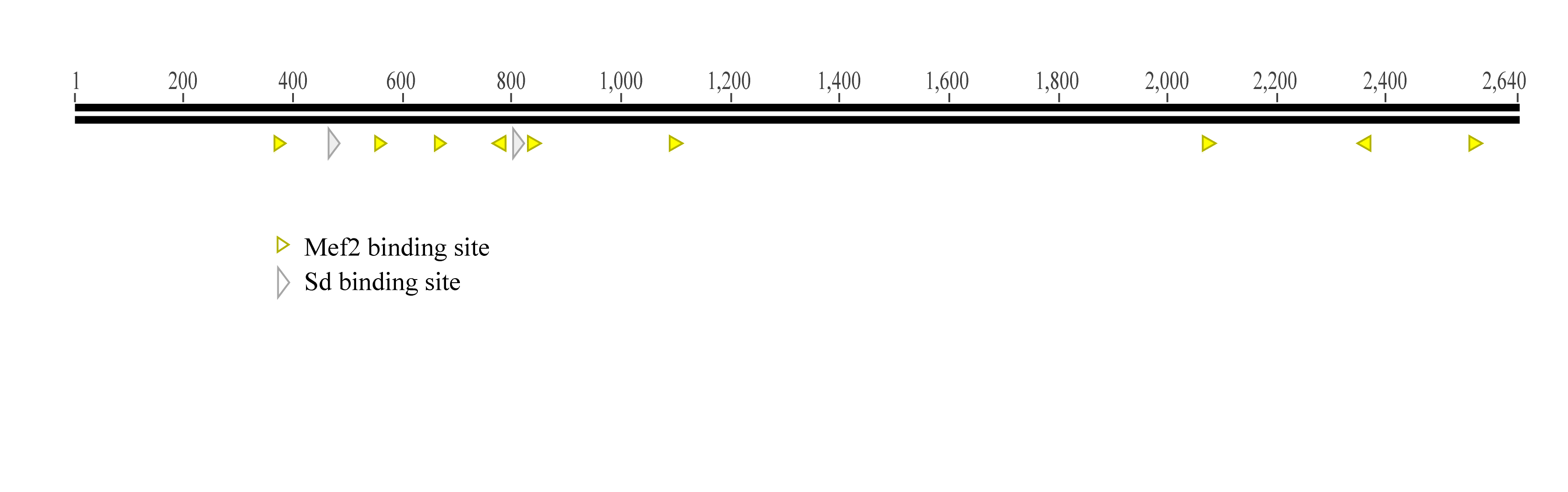

Supplement: Figure S4 — Schematic representation of the Dl2.6 enhancer (3R: 15,142,950..15,145,589). Predicted Mef2 binding sites are shown as yellow arrowheads. Predicted Sd binding sites are shown as gray arrowheads. (TIF) [file pone.0108149.s004.tif]

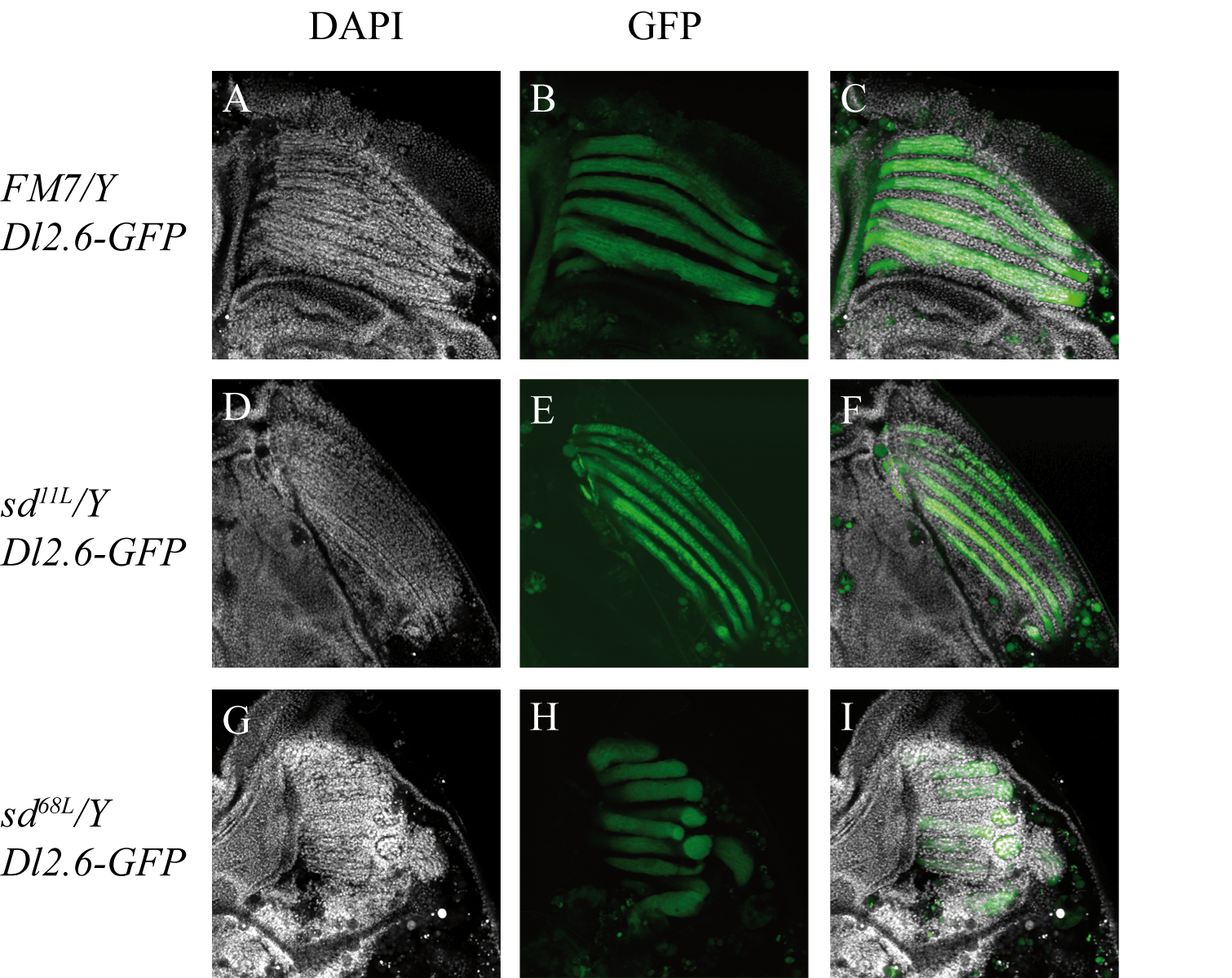

Supplement: Figure S5 — Dl2.6 activation in sd11L and sd68L mutants. In 21 h APF wild-type male pupae (FM7/Y; Dl2.6-GFP/+), the Dl2.6 enhancer is activated in developing IFMs (B, DAPI in A, overlay in C). In 21 h APF sd11L and sd68L male pupae (respectively FM7/sd11L; Dl2.6-GFP/+ and FM7/sd68L; Dl2.6-GFP/+), the Dl2.6 enhancer is also activated (respectively E and H; DAPI in D, G; overlays in F, I). (TIF) [file pone.0108149.s005.tif]
